# Hetero‐Functionalization of Carbon Nanotubes Termini with Single‐Molecule Control

**DOI:** 10.1002/smll.202505186

**Published:** 2025-08-02

**Authors:** Weiying Hong, Benjamin Lambert, Zechariah Mengrani, Laurent Cognet, Matteo Palma

**Affiliations:** ^1^ Department of Chemistry Queen Mary University of London London E1 4NS UK; ^2^ Laboratoire Photonique Numérique et Nanosciences Université de Bordeaux, Institut d'Optique Graduate School, CNRS ‐ UMR5298 Talence 33400 France

**Keywords:** asymmetric synthesis, nanoparticles, nanostructures, nanotubes, synthesis design

## Abstract

A strategy is presented for the asymmetric chemical functionalization of individual single‐walled carbon nanotubes (SWCNTs) termini and their selective conjugation to distinct single moieties, i.e., the formation of bi‐functionalized R‐CNT‐R’ heterostructures. This study demonstrates this via the selective covalent attachment of metal nanoparticles (NPs) and semiconductor nanocrystals (quantum dots, QDs) at opposite ends of individual SWCNTs; the electronic coupling is further characterized between the distinct components of the hybrids. The general applicability of the methodology developed by this study can be beneficial for the design and development of novel SWCNT‐based nanohybrid materials with single‐molecule control.

## Graphical abstract

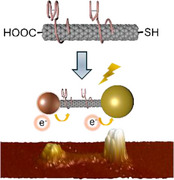

## 1 Introduction

Single‐walled carbon nanotubes (SWCNTs) have shown exceptional potential across a variety of fields, including bioimaging,^[^ ^1^, ^2^, ^3^, ^4^ ^]^ (bio)sensing,^[^ ^5^, ^6^, ^7^, ^8^, ^9^, ^10^, ^11^, ^12^ ^]^ optoelectronics,^[^ ^13^, ^14^, ^15^, ^16^, ^17^, ^18^, ^19^ ^]^ and photocatalysis,^[^ ^20^ ^]^ due to their unique structural, electronic, and optical properties. Site‐specific functionalization of SWCNTs is crucial for applications requiring precise control at the nanoscale, as it enables the integration of single‐molecules,^[^ ^21^, ^22^, ^23^, ^24^, ^25^, ^26^, ^27^, ^28^, ^29^, ^30^ ^]^ defects,^[^ ^31^, ^32^, ^33^, ^34^, ^35^ ^]^ and lattice remodeling in nanotubes.^[^ ^36^ ^]^ These in turn allow for the introduction of specific functions, as well as the modulation and enhancement of the nanotubes' optoelectronic properties, with implications in their optimal use for both optoelectronic and biological applications.^[^ ^37^, ^38^, ^39^, ^40^, ^41^, ^42^, ^43^, ^44^ ^]^ However, existing site‐specific functionalization methods are often limited to single‐type modifications, which restrict the ability to create multifunctional heterostructures with ad‐hoc properties.

In this regard, the asymmetric functionalization of carbon nanotubes (CNT) has been achieved in only a limited number of studies,^[^ ^45^, ^46^, ^47^, ^48^, ^49^, ^50^ ^]^ typically on the sidewalls of the nanotubes, and notably without single‐molecule control when the functionalization was tailored to different CNT termini. So far, asymmetric functionalization of carbon nanotubes termini has been achieved through distinct strategies, such as: i) interfacial inversion of pre‐assembled CNT film, where functional moieties are selectively introduced onto exposed nanotubes surfaces or termini via spatial confinement;^[^ ^48^, ^50^ ^]^ ii) electrochemical polarization, as bipolar electrochemistry induces distinct chemical environments at the CNT termini to drive site selective metal aggregation;^[^ ^51^, ^52^ ^]^ and iii) protection–deprotection based chemistry, where one terminus of the CNT is chemically masked to enable subsequent selective modification at the unprotected end.^[^ ^49^, ^53^ ^]^ While these methods demonstrate the asymmetric functionalization of CNT films or bundles, achieving precise and single‐molecule control at the termini of individual SWCNTs remains a significant challenge This could allow for molecular‐level control in the assembly of carbon nanotubes into novel functional architectures, and would be especially attractive if achieved via an in‐solution methodology toward the development of solution‐processable devices.

Herein, we report an aqueous solution strategy for the asymmetric chemical functionalization of individual SWCNTs termini and their selective conjugation to distinct single nanoscale moieties, offering molecular‐level spatial control. As proof of concept, the formation of bi‐functionalized R‐CNT‐R′ heterostructures was demonstrated via the selective covalent attachment of metal nanoparticles (NPs) and semiconductor nanocrystals (quantum dots, QDs) at opposite ends of individual SWCNTs. We further characterized the electronic coupling between the components of the hybrids via photoluminescence (PL) spectroscopy. The control and general applicability of the methodology we developed can be beneficial for the design of novel SWCNT‐based nanohybrid materials, in particular for optoelectronic applications.

## 2 Result and Discussion

**Scheme 1:**
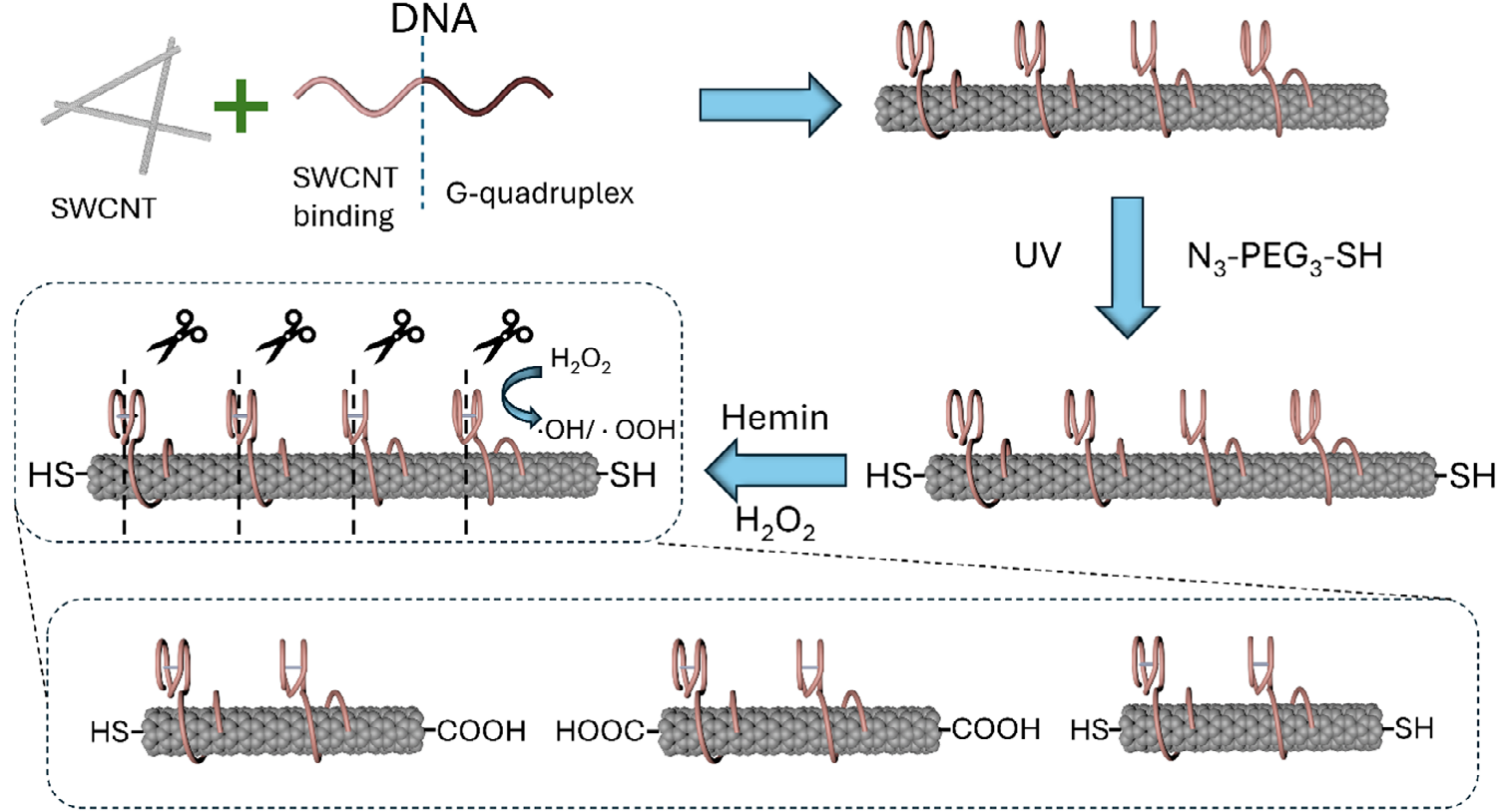
The asymmetric chemical functionalization of SWCNTs termini via the i) nanotubes wrapping with a G‐quadruplex sequence, ii) their thiol end‐functionalization, and iii) the nanotubes oxidative cutting by hemin and H_2_O_2_.

We employed (6,5) chirality‐enriched semiconducting SWCNTs wrapped with single‐stranded DNA (ssDNA) [see the Supporting Information (SI)]. DNA wrapping^[^ ^54^, ^55^ ^]^ grants dispersion of nanotubes in aqueous solution, and here we designed it to feature two functional domains: a (6,5) SWCNT affinity domain that binds and protects their sidewalls for end‐specific functionalization,^[^ ^24^, ^25^, ^27^, ^28^, ^56^, ^57^, ^58^ ^]^ and a G‐quadruplex domain that confines hemin for subsequent localized oxidative cutting of the nanotubes.^[^ ^59^ ^]^ Our hetero‐functionalization strategy (see **Scheme** **1** and the Supporting Information) begins with the introduction of a thiol functionality to the SWCNTs termini, achieved by tethering a short azide‐modified polyethylene glycol thiol (N_3_‐PEG_3_‐SH) to the sidewall‐protected nanotubes via a UV‐induced cycloaddition reaction.^[^ ^27^ ^]^ Subsequently, hemin was introduced and anchored along the SWCNTs sidewalls via the G‐quadruplex domain of the DNA‐wrapping sequence, providing spatial control over the oxidation sites on the nanotubes: hemin, with its peroxidase‐like activity, catalyzes the conversion of hydrogen peroxide into reactive oxygen species, enabling site‐specific oxidative cutting of SWCNTs.^[^ ^59^ ^]^ This allowed us to generate new carboxylic acid termini on the SWCNTs, while preserving the sidewall integrity of SWCNTs in aqueous solution and at least part of the thiol end‐functionalization introduced in the first step, as only small amounts of hemin and H_2_O_2_ were required. It is reasonable to assume that this process results in a mixture of symmetric and asymmetric end‐functionalized SWCNTs, i.e., thiol‐SWCNT‐thiol and thiol‐SWCNT‐COOH segments, as shown in Scheme 1.

We confirmed the aforementioned thiol‐functionalization and oxidative cutting of the SWCNTs by tethering gold nanoparticles (AuNPs) to the nanotubes termini, both before and after the cutting. Atomic Force Microscopy (AFM) imaging of solutions cast on mica revealed star‐like assemblies where each AuNP was tethered to more than one SWCNT (Figure S1, Supporting Information), a behavior likely due to the large (20 nm) AuNPs size compared to the ≈2 nm nanotubes diameter. Most thiol‐functionalized and cut SWCNTs were found to be tethered to a AuNP only at one of their termini (≈82 %), while 18% of the observed SWCNTs were bound to AuNPs on both ends of the nanotube (Figure S1d, Supporting Information). When smaller AuNPs (5 nm, Figure S2, Supporting Information) were used, fewer SWCNTs were found to bind to the same nanoparticle. This is likely due to the reduced surface area and higher curvature of the smaller AuNPs, which limit the number of available binding sites for multiple SWCNTs—a phenomenon also observed in other AuNP‐based systems.^[^ ^60^ ^]^ Additionally, we observed a significant increase in the radial breathing mode (RBM) region at 290 cm^−1^ (Figure S3, Supporting Information), characteristic of (6,5) SWCNTs Raman spectra.^[^ ^61^ ^]^ This enhancement aligns with previous observations in Au‐nanorod–SWCNT heterostructures, where metal growth induced notable changes in Raman features, including RBM amplification due to reduced bundling and improved nanotube isolation^[^ ^62^ ^]^ Localized surface plasmon resonance (LSPR) effects from the AuNPs may contribute to this enhancement, as supported by the observed spectral overlap between the excitation laser and the AuNP absorption band in the UV–vis data (Figure S4, Supporting Information). The selective increase of the (6,5) RBM signal further reflects the use of a DNA wrapping sequence (TTT ATT ATT ATT AT) known for its high affinity and selectivity toward (6,5) SWCNTs,^[^ ^63^ ^]^ supporting the successful dispersion and stabilization of individualized nanotubes in our system. The nanotube cutting was quantitatively confirmed by AFM length analysis of the SWCNTs before and after the oxidative process (Figures S5 and S6, Supporting Information). The median length of the SWCNTs decreased from 441 nm for the starting solution, to 272 nm after oxidation, indicating that most nanotubes were cut into two segments.

**Figure 1:**
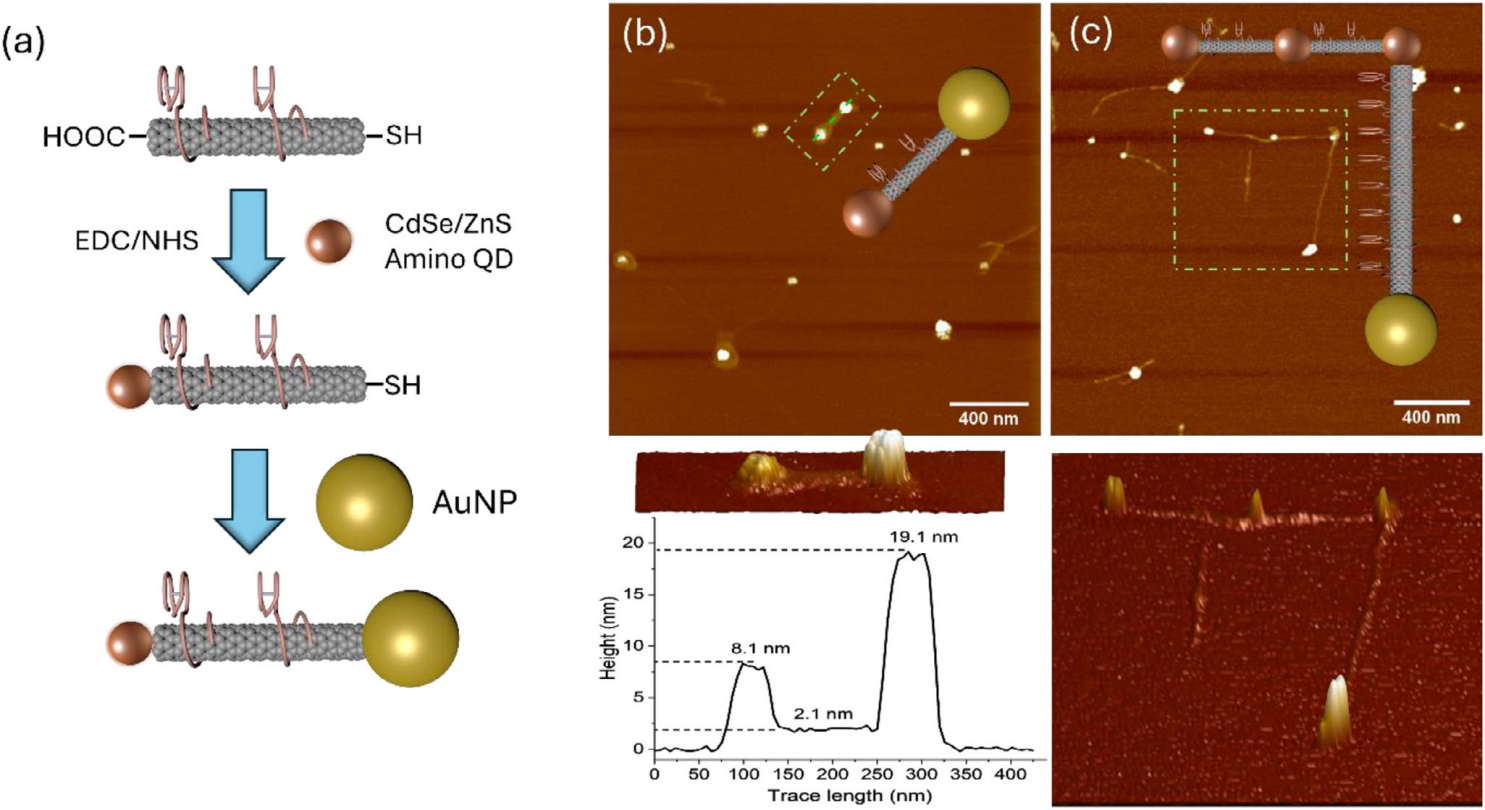
Schematic of the assembly steps for the formation of SWCNT nanohybrids. Representative topographical AFM images of b) 1:1:1 Au‐SWCNT‐QD heterostructure, together with a height profile, and c) linear and branched SWCNT‐NP junctions (Z‐scale=20 nm). The corresponding 3D plots of the areas in the green dashed boxes are displayed directly below each 2D contour.

In order to further demonstrate the asymmetric end‐functionalization of the aforementioned thiol‐functionalized and cut SWCNTs segments, we aimed at tethering amino QDs (≈8 nm) to the carboxylic groups present on the SWCNTs termini (Figure S7, Supporting Information) as we previously shown,^[^ ^23^ ^]^ and AuNPs to the thiol terminating ends of the nanotubes (**Figure** **1**a). We observed, via AFM, the attachment of at least one NP to the termini of ≈72% of the SWCNTs present in the mixture (Figure S8, Supporting Information). Among these nanohybrids, the yield of formation of AuNP‐SWCNTs‐QDs heterostructures was found to be ≈13%, where both 1:1:1 heterostructures (Figure 1b; Figures S8a and S9, Supporting Information) and linear as well as branched SWCNT junctions (Figure 1c; Figures S8b and S9, Supporting Information) were observed. As expected, most of the SWCNT nanohybrids presented QDs to their termini, typically via nanotubes junction formation; this is due to the overall greater presence of carboxylic acid terminal functionalities on the nanotubes segments (see Scheme 1). This structural distribution was determined from statistical analysis of hundreds of individual SWCNTs in the hybrid samples, providing representative quantification of the various nanostructure configurations (see the Supporting Information).

To further explore the platform's ability to accommodate diverse terminal components, we assembled GFP–SWCNT–QD heterostructures (see the Supporting Information). Notably, these nanohybrids exhibited a higher proportion of well‐defined 1:1:1 architectures, which we attribute to the smaller size (≈2 nm) and the defined binding interface of GFP (Figure S10, Supporting Information). Overall nanoparticle conjugation conditions, such as concentrations and moiety ratios, were optimized in the construction of all our hetero‐functionalized SWNCT nanohybrids: see Tables S1–S4 and Figures S11–S13 (Supporting Information). To highlight the distinct advantages of our solution‐phase approach, we summarized a comparison of representative asymmetric functionalization strategies across key parameters—including site specificity, scalability, and single‐molecule control capability—in Table S5 (Supporting Information).

In order to probe the coupling and decay dynamics of the electronic excitations between the SWCNTs and the nanoparticles in the hybrids, we performed PL spectroscopy investigations. Moreover, we replaced DNA wrapping with 1% sodium deoxycholate (DOC) after hybrid formation,^[^ ^64^ ^]^ so to minimize potential non‐specific interactions between SWCNTs and the NPs. Additionally, we employed 5 nm AuNPs that, as mentioned earlier, mitigate excessive network formation in SWCNT‐AuNP heterostructures (Figure S1, Supporting Information).

**Figure 2:**
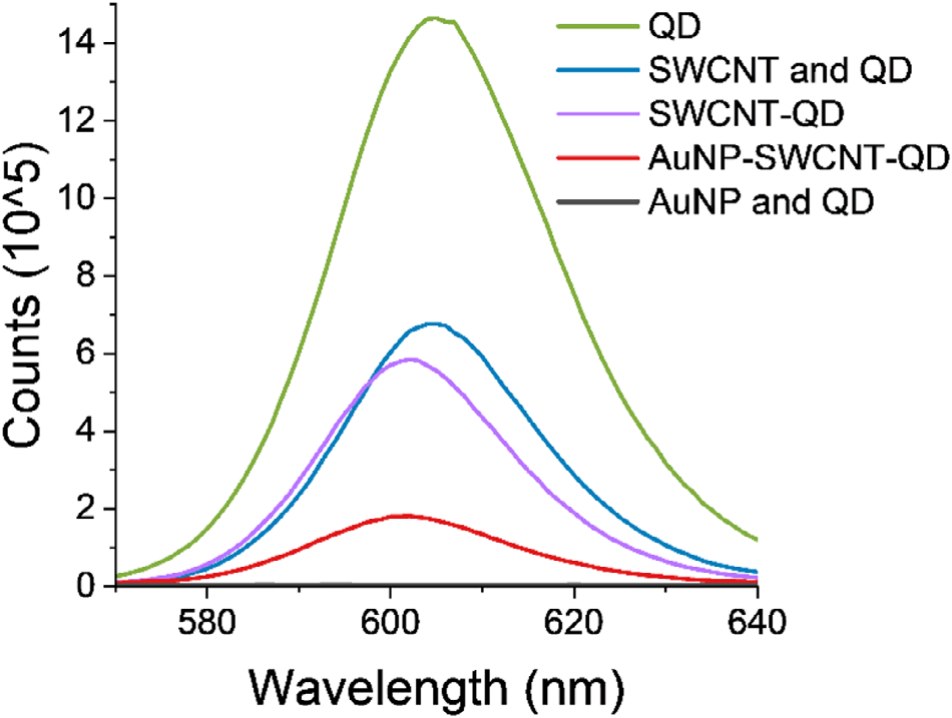
Steady‐state photoluminescence characterization of QD (green), SWCNT‐QD hybrids (purple), AuNP‐SWCNT‐QD (red), control samples are the SWCNT mixed with QD without the formation of heterostructures (blue) and Au mixed with QD (black), excited under 450 nm.

Steady‐state PL (SSPL) measurements revealed a 60% fluorescence quenching and blue‐shift for the QDs emission in the QD‐SWCNT hybrids, compared to the pristine QDs emission (**Figure** **2**), as previously observed in various QD‐CNT hybrids.^[^ ^65^, ^66^ ^]^ Control PL spectra of individual components and simple mixtures are shown in Figure S14 (Supporting Information), confirming that the observed effects arise from the designed heterostructures. These results are strongly indicative of electronic coupling between the SWCNTs and the QDs in the hybrids, as we have previously shown.^[^ ^23^ ^]^ The quenching in these covalently linked SWCNT‐QD heterostructures was found to be 6% greater than the one observed for a simple mixture of QDs and SWCNTs, where physisorption can still induce a degree of coupling between the nanohybrid components. The AuNP‐SWCNT‐QD heterostructures displayed a quenching efficiency of up to ≈87% compared to pristine QDs PL, likely due to QD‐AuNP interactions within the solution from increased nonradiative decay pathways, as previously shown;^[^ ^67^ ^]^ this effect is also evident by the close to complete quenching of QDs emission when simply mixed with AuNPs (Figure 2). To support this interpretation, dynamic light scattering (DLS) measurements (Figure S15, Supporting Information) show that the hydrodynamic diameter of QDs alone is centered ≈1.8 nm, while that of AuNPs is ≈7.1 nm. Notably, the mixed QD–AuNP solution exhibits a broadened size distribution with a central value of ≈9.7 nm, indicating partial aggregation.

**Figure 3:**
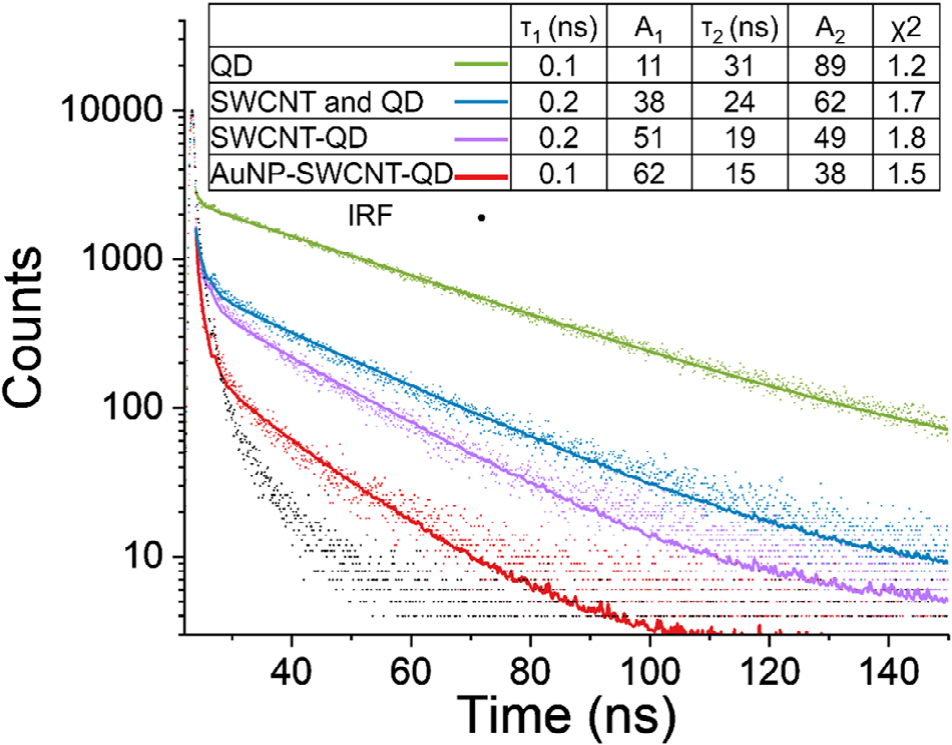
TRPL characterization of QD (green), SWCNT mixed with QD (blue), SWCNT‐QD hybrids (purple), AuNP‐SWCNT‐QD (red) with the corresponding fitting curve, and IRF in black. The inset shows the table of the TRPL parameters extracted from the PL curves.

Time‐resolved PL (TRPL) measurements were carried out to study the decay dynamics of the electronic excitations in our heterostructures, and deduce the coupling mechanism between the nanostructures forming the hybrids. **Figure** **3** shows that pristine QDs exhibit lifetime decay with a biexponential trace, in agreement with previous studies on QDs and their hybrids.^[^ ^23^, ^26^, ^68^ ^]^ Within this fitting model (see the Supporting Information) the shorter lifetime (τ_1_) indicates the fast decay attributed to core‐state recombination, and the long lifetime (τ_2_) represents the slow decay attributed to surface states and trap states, while A_1_ and A_2_ are the pre‐exponential factors corresponding to these decay components. In the SWCNT‐QD hybrids, the shortening of the longer lifetime (τ_2_) of the QDs emission accompanied by an increase in the A_1_, is indicative of a shift toward monoexponential decay, in line with evidence of charge transfer between the nanoparticle and the nanotube, as we and others have shown.^[^ ^23^, ^26^, ^65^, ^69^ ^]^ This trend was even greater in the AuNP‐SWCNT‐QD hybrids, where τ_2_ decreased to 15 ns, and A_1_ increased from 11% to 62% (Figure 3). The shortened τ_2_ and transition toward a mono‐exponential‐like decay implies that surface states (τ_2_) no longer significantly contribute to the QDs emission, suggesting an enhanced electronic coupling in the presence of AuNP and SWCNTs. These results indicate charge transfer is likely the dominating coupling effect between the QDs and the nanotubes in all the terminally linked SWCNT‐QD hybrids. This interpretation is further corroborated by the data presented in Figure S16 (Supporting Information). Although spectral overlap exists between the QD emission and SWCNT excitation (Figure S16a, Supporting Information), the quenching of QD's fluorescence observed in both the hybrids and the mixed control (Figure S16b, Supporting Information), combined with the fact that SWCNT PL enhancement occurs only in the covalently linked hybrids (Figure S16c, Supporting Information), suggests that photoinduced energy transfer is not a significant contributor in this system. Instead, the PL modulation observed in the hybrids likley arises primarily from charge transfer processes.

**Figure 4:**
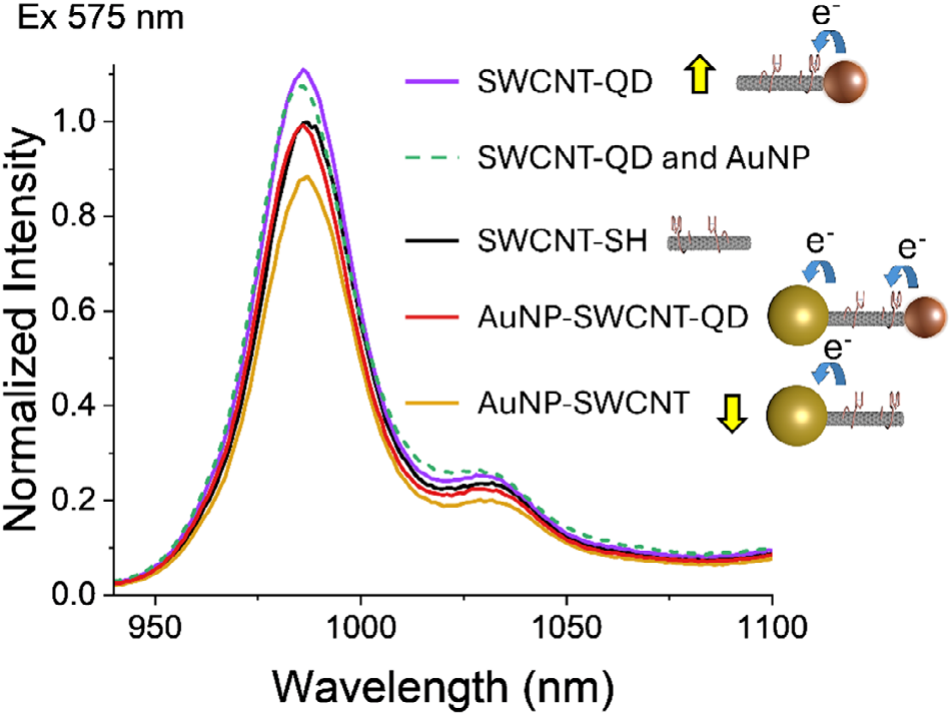
Steady‐state photoluminescence characterization of SWCNT‐based hybrids under 575 nm excitation for hetero‐end‐functionalized SWCNT‐QD hybrids (purple), control Au mixed with QD‐SWCNT hybrids (green dotted line), SWCNTs (black), AuNP‐SWCNT‐QD (red), and AuNP‐SWCNT (yellow) hybrids.

To further investigate the overall electronic coupling occurring in all our heterostructures, we monitored the PL changes of SWCNTs under 575 nm to match the (6,5) SWCNTs excitation (**Figure** **4**), control PL spectra of individual components are shown in Figure S17 (Supporting Information). Mono‐functionalized QD‐ and AuNP‐SWCNT show 11% enhancement and 12% quenching of the nanotubes PL, respectively (Figure 4). These opposing trends suggest that QDs and AuNPs interact with SWCNTs via distinct electronic mechanisms. Such effects were not observed under 845 nm excitation (Figure S18, Supporting Information), where the excitation of QDs and resonance of AuNPs were avoided.

An enhancement of the PL of the SWCNTs in the presence of QDs compared to the thiol functionalized SWCNT after cutting, is attributable to an excited‐state electron transfer from the nanoparticle conduction band (CB) to the CB of nanotube.^[^ ^70^, ^71^ ^]^ This mechanism has been previously observed in various nanohybrid systems^[^ ^68^, ^69^, ^72^ ^]^ and is facilitated by favorable band alignment between CdSe/ZnS QDs^[^ ^72^ ^]^ and SWCNTs^[^ ^73^ ^]^ (Figure S19, Supporting Information), enabling efficient charge transfer. This transient electron injection counteracts hole‐doping effects commonly induced by environmental oxygen under ambient conditions, thereby enhancing the radiative recombination within the SWCNTs and increasing their PL intensity. While similar PL modulation has been reported in the context of reducing agents^[^ ^74^ ^]^ and pH‐induced changes,^[^ ^75^ ^]^ in our system the enhancement is driven by an excitation‐dependent charge transfer process. The observed SWCNTs PL enhancement (11%) is not as significant as the QDs quenching (60%, Figure 2), likely due to the relatively minor contribution of nanotubes termini to the overall SWCNTs PL.^[^ ^76^ ^]^

Upon mono‐conjugation with AuNPs, the PL of SWCNTs exhibited 12% quenching compared to thiol‐functionalized SWCNTs after cutting. Raman spectroscopy further confirmed that this quenching is not due to structural damage, as no significant changes were observed on the D/G ratio after AuNP conjugation (Figure S20, Supporting Information). While LSPR effects from AuNPs may be present — as supported by the spectral overlap between the AuNP plasmon band and the SWCNT E_22_ excitation wavelength (Figure S21, Supporting Information) — the rapid relaxation from the E_22_ to the lower‐energy E₁₁ exciton would typically suggest that any plasmonic enhancement should manifest in the E₁₁ photoluminescence. However, we did not observe any PL enhancement typically associated with plasmonic coupling in the AuNP‐SWCNT nanohybrids.^[^ ^77^ ^]^ Electron withdrawing from the nanotubes has been found to cause SWCNTs PL quenching,^[^ ^78^, ^79^ ^]^ and metal nanomaterials have shown electron‐withdrawing effects on SWCNTs.^[^ ^80^ ^]^ In our system, the covalent attachment of AuNPs at the termini provides a direct pathway for electron transfer. The use of DOC further minimizes nonspecific sidewall interactions, allowing us to attribute the observed PL suppression primarily to terminal electron withdrawal, consistent with the band alignment between AuNP and SWCNTs.^[^ ^73^, ^81^ ^]^

Building on the distinct PL behaviors observed in QD‐SWCNT and AuNP‐SWCNT hybrids, we next examined the AuNP‐SWCNT‐QD hybrids system. As shown in Figure 4, the SWCNTs PL intensity of AuNP‐SWCNT‐QD heterostructures was found to be lower than the QD‐SWCNT hybrids but greater than the AuNP‐SWCNT structures. This suggests a balance between the electron injection from the QDs and the electron withdrawal by AuNPs in the AuNP‐SWCNT‐QD heterostructures. Notably, simple mixtures of AuNPs with QD‐SWCNT hybrids exhibited a PL intensity comparable to the QD‐SWCNT nanohybrids (Figure 4), highlighting the key role played by AuNPs attachment to the SWCNTs termini in the PL quenching observed in the AuNP‐SWCNT‐QDs heterostructures.

To rationalize the photophysical behavior observed in our AuNP‐SWCNT‐QD hybrid heterostructures, we correlated the aforementioned SWCNT PL data (Figure 4) with the nanoparticle attachment yield as calculated from AFM imaging for all the hybrid structures (Figure S8c, Supporting Information). The PL responses of all SWCNT hybrids samples were normalized to that of the thiol‐functionalized SWCNT after cutting. For each kind of SWCNT hybrid (e.g., QD‐SWCNT versus AuNP‐SWCNT), we employed the overall attachment yield, i.e., grouping the 1:1, 2:1, and sidewall attachment together. The ≈37% yield of formation of QD‐SWCNT hybrids (Figure S7, Supporting Information), resulted in an 11% increase in SWCNT's PL intensity relative to the starting SWCNTs. The contribution of QD‐SWCNT hybrids assuming a 100% yield to the PL enhancement was then calculated to be 11%/37% = 30%. Similarly, for AuNP‐SWCNT hybrids (≈23% yield of formation and 12% quenching), the relative PL contribution for a 100% yield was determined to be −12%/23% =−52%, i.e., a 52% quenching to the SWCNTs PL. For the AuNP‐SWCNT‐QD heterostructures, we considered the yields of both QD‐SWCNT (≈53%) and AuNP‐SWCNT (≈42%) interfaces in the formed nanohybrids (Figure S8c, Supporting Information). We can therefore estimate the SWCNTs PL intensity change for the AuNP‐SWCNT‐QD solutions as (30%  ×  53%)−(52%  ×  42%)=−6%, i.e., resulting in an overall 6% quenching to SWCNT PL. This is broadly in agreement with the 2% quenching we observe in our PL investigations (red vs black line in Figure 4). By and large, our overall findings suggest a balance of competing electron‐donating and withdrawing effects by the QDs and AuNPs, respectively, when tethered to distinct SWCNT termini.

## 3 Conclusion

In summary, we established an approach for the in‐solution asymmetric chemical functionalization of individual SWCNTs termini, toward single‐molecule control. AFM imaging confirmed the formation of well‐defined hybrid heterostructures. Photoluminescence investigations highlighted the interplay between differing charge transfer processes at the opposite termini of the nanotubes in the assembled AuNP‐SWCNT‐QD heterostructures, with electron transfer occurring from the QDs to the SWCNTs, and electron withdrawing by the AuNPs. The strategy we presented is of general applicability for the construction of single‐molecule hetero‐junctions with controlled charge carrier pathways, toward a variety of potential applications in optoelectronics^[^ ^13^, ^14^, ^15^, ^37^, ^82^ ^,]^ and photocatalysis.^[^ ^83^, ^84^ ^]^ For example, terminal AuNP conjugation could facilitate direct interfacing with electronic platforms, enabling the fabrication of single‐molecule electronic junctions. Moreover, while the current study is a proof‐of‐concept, the strategy is inherently compatible with scalable solution‐phase chemistries; future efforts will focus on application‐oriented optimizations, including scaling up reaction throughput, improving conjugation efficiency, and establishing structure–function relationships tailored to specific optoelectronic or sensing applications.

## Conflict of Interest

The authors declare no conflict of interest.

## Supporting information



Supporting Information

## Data Availability

The data that support the findings of this study are available in the supplementary material of this article.
