# Supplementary material for: Hetero‐Functionalization of Carbon Nanotubes Termini with Single‐Molecule Control
Source: Small. 2025 Aug 2;21(38):e05186. doi: 10.1002/smll.202505186 (PMC12462589; doi:10.1002/smll.202505186)
Supplement: Supplementary file 1 — Supporting Information [file SMLL-21-e05186-s001.docx]

Supporting Information

Hetero-Functionalization of Carbon Nanotubes Termini with Single-Molecule Control

Weiying Hong, Benjamin Lambert, Zechariah Mengrani, Laurent Cognet,* and Matteo Palma*

Dr W. Hong, Z. Mengrani, Prof. M. Palma

Department of Chemistry

Queen Mary University of London

London, E1 4NS, UK
E-mail: m.palma@qmul.ac.uk

Dr B. Lambert, Prof. L. Cognet
Laboratoire Photonique Numérique et Nanosciences

Université de Bordeaux

Institut d'Optique Graduate School, CNRS - UMR5298

Talence, 33400, France

E-mail: laurent.cognet@u-bordeaux.fr

**Materials**

All oligonucleotide syntheses were obtained from IDT. SWCNT (6,5) chirality (773735), CdSe/ZnS QD (919322, emission 600 nm), Sodium deoxycholate (DOC, D6750, Cas 302-96-4) and AuNP (5nm, 741949/20nm, 741965) were purchased from Sigma-Aldrich. Thiol-PEG3-azide (CAS: 1347750-79-1) was purchased from Conju-Probe, dissolved in anhydrous methanol to a concentration of 0.1M as a stock solution, and kept in the freezer. Tris-borate-EDTA (TBE) buffer 10X and H_2_O_2_ (30% in water, BP2633, Cas 7732-18-5) were purchased from Thermo Fisher. Hemin (H9039, Cas16009-13-5) was purchased from Scientific Laboratory Supplies. 1-(3-Dimethylaminopropyl)-3-ethylcarbodiimide hydrochloride (EDC, A10807.6, Cas 25952-53-8) was purchased from Alfa Aesar. Sulfo-N-hydroxysulfosuccinimide sodium salt (sulfo-NHS, H1304, Cas 106627-54-7) was purchased from TCI.

**SWCNT wrapping**

SWCNTs were dispersed by wrapping with a DNA sequence (GTG GGT AGG GCG GGT TGG TTT ATT ATT ATT AT), where the GTG GGT AGG GCG GGT TGG segment forms a G-quadruplex, while the TTT ATT ATT ATT AT segment facilitates wrapping around the SWCNT sidewall. To prepare the DNA-wrapped SWCNTs, 1mg of SWCNT was mixed with 2.5 mg of DNA in 1 mL of 100 mM NaCl. The mixture was subjected to bath sonication (Branson 2800) for 90 minutes. After sonication, the dispersion was centrifuged at 16,000 g for 30 minutes and 80% of the supernatant was carefully collected. The concentration was calculated as 1Abs_990_=13 µg/mL.

**Thiol-SWCNT end-functionalization**

The reaction solution was prepared by combining 27.8 µg SWCNT in 30% methanol and adding 10 µL thiol-PEG_3_-N_3_ (0.1 M). The mixture was irradiated using a handheld UV lamp (UVM-57, 6 W, 302 nm) for 20 minutes and then incubated overnight at room temperature (R.T.). Following incubation, MgCl_2_ was added to the reaction solution to achieve a final concentration of 25 mM, promoting precipitation of the functionalized SWCNTs. The solution was centrifuged at 7600 g for 5 minutes, and the supernatant containing unreacted thiol-PEG_3_-N_3_ and free DNA was discarded. The remaining pellet was resuspended in TBE buffer to adjust the final concentration to 100 µg/mL. A short bath sonication was applied if the solution exhibited aggregation.

**Thiol-SWCNT-carboxyl hetero-end-functionalization**

For forming hetero-end-functionalized SWCNT, a cutting method was adapted from Atsume et al^[1]^. 10 µL of thiol-SWCNT was dissolved in 40 µL Tris-HCl (20 mM KCl 50 mM pH 8) and mixed with 10 µL TBE buffer. The solution was incubated at 70℃ for 10 minutes, followed by gradual cooling to 15 ℃ over 55 minutes at a controlled rate of -1^o^C per minute to promote wrapping DNA sequence annealing. 1 µL of 250 µM hemin dissolved in DMSO was diluted to 2.5 pM, 0.4 µL of this dilution was added to the annealed solution and incubated for 1 hour at R.T. to facilitate hemin binding to G-quadruplex structure. Following this, 0.4 µL of 1% hydrogen peroxide was introduced to the sample, which was further incubated at room temperature overnight to induce oxidative cutting of the SWCNT. This process generated SWCNTs with two distinct terminal functional groups, enabling the construction of dual-end-functionalized SWCNTs for advanced applications.

**Assembly of the Au-SWCNT-QD hybrids**

The dual-end-functionalized SWCNT solution was mixed with 2 µL of a freshly prepared solution containing 100 mM EDC and 50 mM sulfo-NHS in Tris buffer (pH 8.0) with 50 mM KCl. Subsequently, 0.8 µL of 1mg/mL QDs were added to the SWCNT solution, and the mixture was incubated at R.T. for 2 hours, to allow for the coupling reaction. Following the incubation, 40 µL of Tris-HCl buffer (pH 8.0) containing 50 mM KCl was added to quench the amidation reaction. Finally, 5 µL of 5 nm AuNPs (OD 40) were introduced to the mixture to facilitate the formation of the tri-component heterostructure.

**Optical characterization techniques (Raman, UV-Vis, PL Spectrum)**

Raman spectroscopy was performed using a Renishaw inVia Raman microscope with a 633 nm laser excitation source. UV-Vis absorption spectra were recorded using a Shimadzu UV-3600 spectrometer. Steady-state photoluminescence (SSPL) and time-resolved photoluminescence (TRPL) measurements were carried out using an Edinburgh Instruments FLS1000 spectrometer with PMT-980 and NIR PMT-1400 detectors. For SSPL spectra, excitation was provided by a 450 W ozone-free Xenon arc lamp, and TRPL decays were recorded using EPL-375 picosecond pulsed laser diodes.

SWCNT, AuNP-SWCNT, SWCNT-QD, and AuNP-SWCNT-QD hybrid nanostructures were measured in solution conditions with 1% DOC to exchange wrapping DNA and maximize fluorescence. To ensure consistency, the concentration of all components was kept identical across the samples and a buffer was added in control samples to maintain uniform conditions.

TRPL decay curves were fitted using a bi-exponential function:

$$I(t)=A_{1}ⅇ^{-t/\tau_{1}}+A_{2}ⅇ^{-t/\tau_{2}}$$

Where A_1_ and A_2_ are the amplitude coefficients, and τ_1_ and τ_2_ represent the respective decay lifetimes associated with different recombination pathways. The quality of the fit was assessed using the reduced chi-square (χ²) statistics, with a value close to 1 indicating a good fit to the experimental data.

**Atomic force microscopy**

AFM was carried out on a Bruker Dimension Icon in Peak Force Tapping mode using ScanAsyst Air tips (Bruker). All AFM images were acquired at a scan rate of 0.977 Hz with a resolution of 512x512 pixels. Sample preparation for AFM imaging of the AuNP-SWCNT-QD nanohybrids followed the method described previously, with adjustments to reduce the concentration and system size. To clearly differentiate between the two 0D materials, 20 nm AuNPs were used in the morphology observation of the hybrid structures. Briefly, 1µL thiol-SWCNT-carboxyl was diluted 20 times into a mixed solution of 16 µL of Tris buffer (pH 8.0) with 50 mM KCl with 4 µL TBE. To facilitate the formation of SWCNT-QD hybrids, 0.6 µL sulfo-NHS/EDC (5mM/10mM) and 0.4 µL 10µg/mL QD were added in the diluted SWCNT solution and incubated for 2 hours at room temperature. 5 µL of the SWCNT-QD solution was mixed with 8.4 µL Tris buffer (pH 8.0) with 50 mM KCl and 3.5 µL 20 nm AuNP (OD1) and incubated at R.T. for 3 min. Freshly cleaved mica discs (Agar Scientific) were prepared by cleaving with adhesive tape, solution of Au-SWCNT-QD tri-component was deposited onto the treated mica and incubated at room temperature for 10 min. Following incubation, the samples were rinsed with 20 µL MilliQ water and dried with compressed air before imaging.

In the calculations of the hybrids yield of formation from AFM imaging, individual SWCNTs were used as the unit of calculation. The presence of nanoparticles at either terminus or along the sidewall was assessed for each nanotube to determine attachment yield. For clarity, junction structures e.g., QD-SWCNT-QD-SWCNT-AuNP were counted as individual NP-SWCNT hybrids, as QD-SWCNT-QD and QD-SWCNT-AuNP included in the yield percentages.


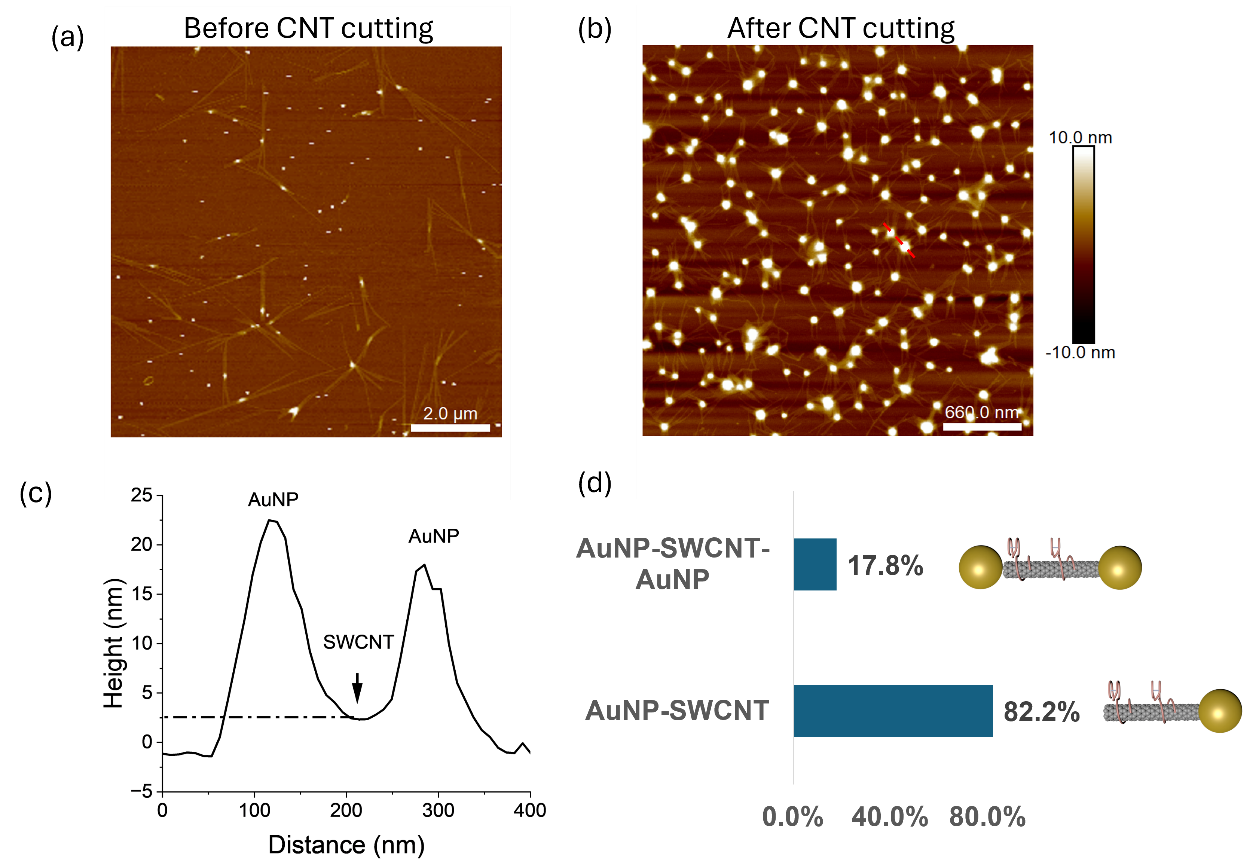


*Figure S1. a) Representative AFM image of hybrids forming from 20 nm AuNPs with SWCNT (a) before and (b) after cutting, accompanied by (c) a height profile graph corresponding to the marked red dash line in the AFM image in (b) (Z-scale=20 nm), and d) Statistical distribution of nanostructures observed in the AuNP-SWCNT hybrids assembly after CNT cutting, obtained from the analysis of 101 individual SWCNTs based on AFM imaging (nanotubes forming junctions were counted as individual NP-SWCNT hybrids).*

*
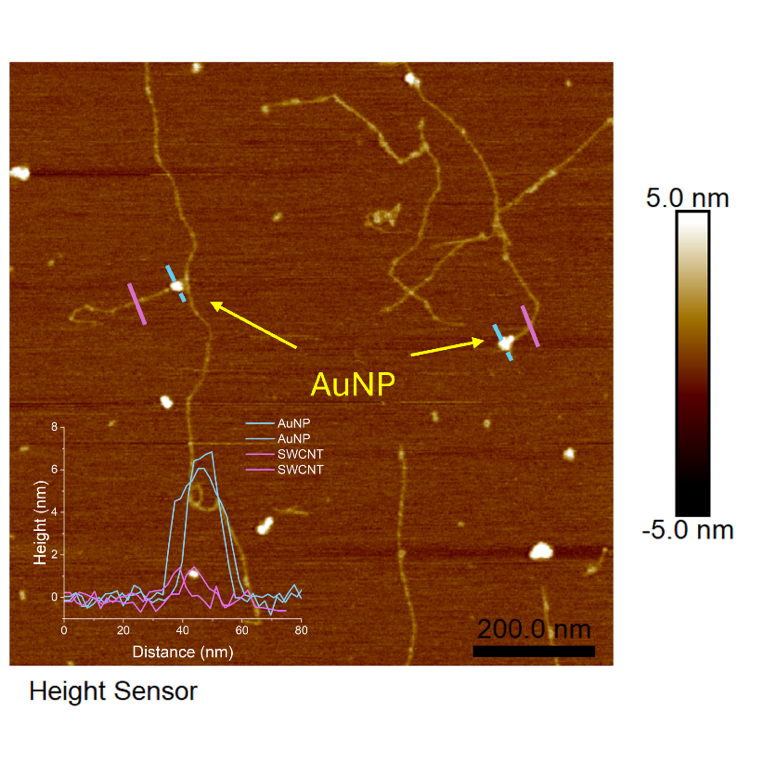
*

*Figure S2. Representative AFM image of AuNP-SWCNT hybrids (AuNP 5 nm) with an inset profile height graph along with the marked line, indicating SWCNT (purple) and AuNP (blue), AuNPs are highlighted by the yellow arrows (Z-scale=10 nm).*


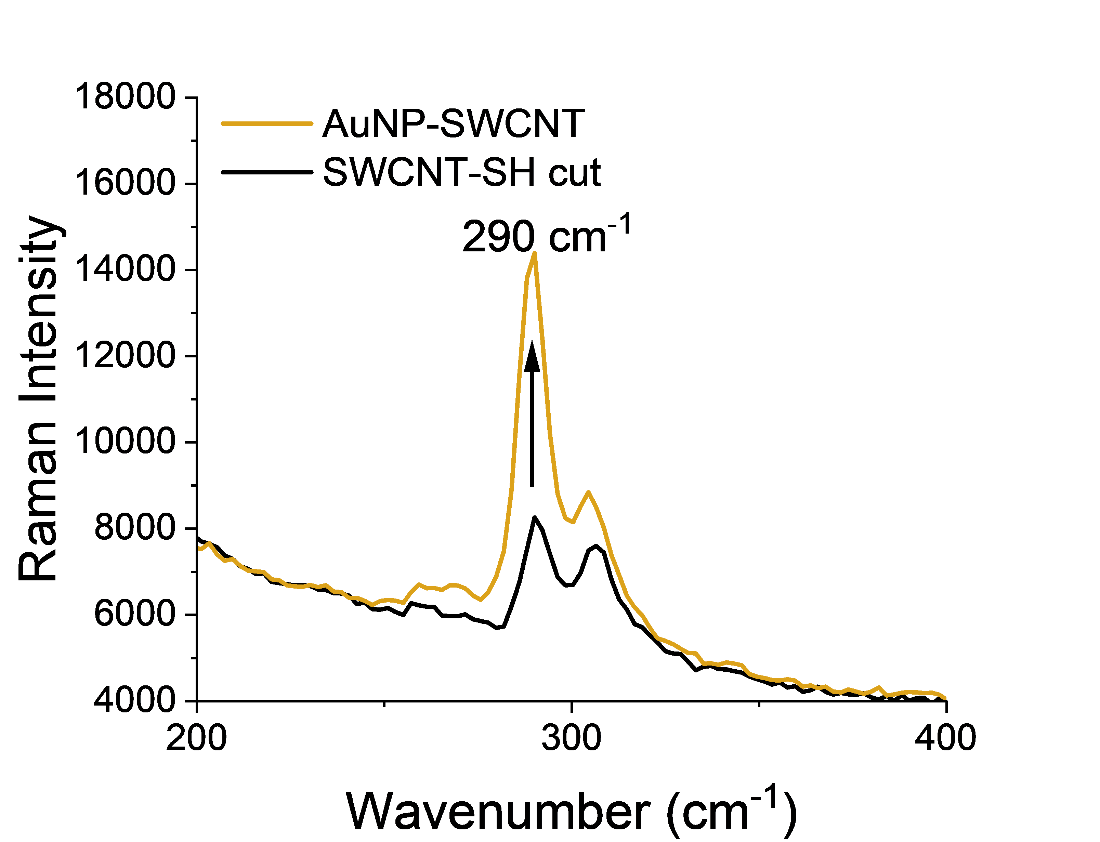


*Figure S3. Raman spectra of thiol functionalized SWCNT after cutting (black), AuNP-SWCNT hybrids (yellow).*

*
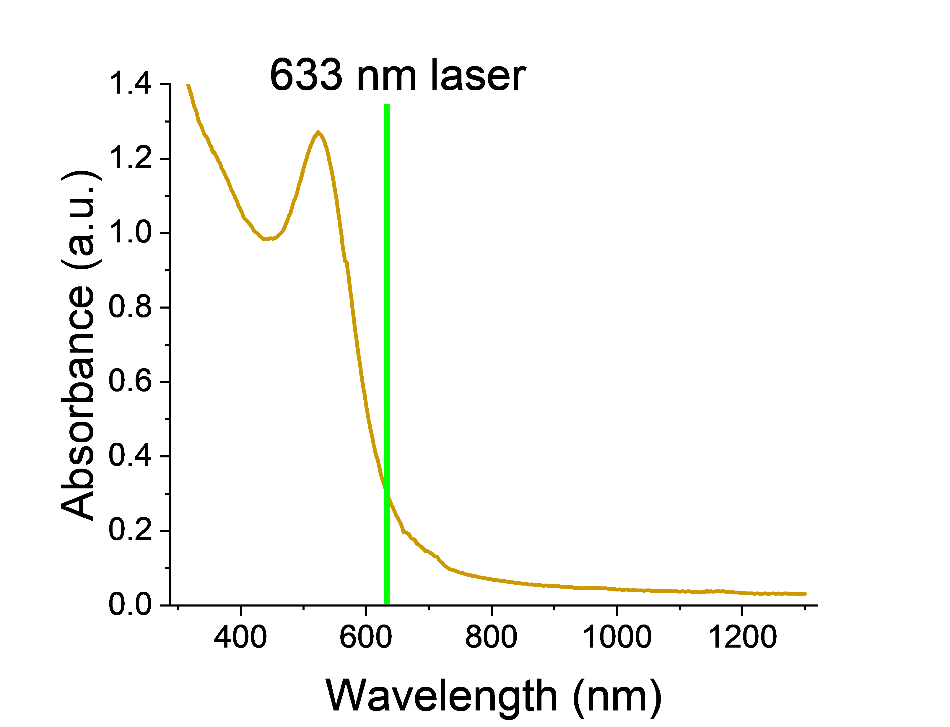
*

Figure S4. UV-Vis absorbance spectrum of AuNPs. The green dashed line indicates the Raman excitation wavelength (633 nm) used in our measurements.


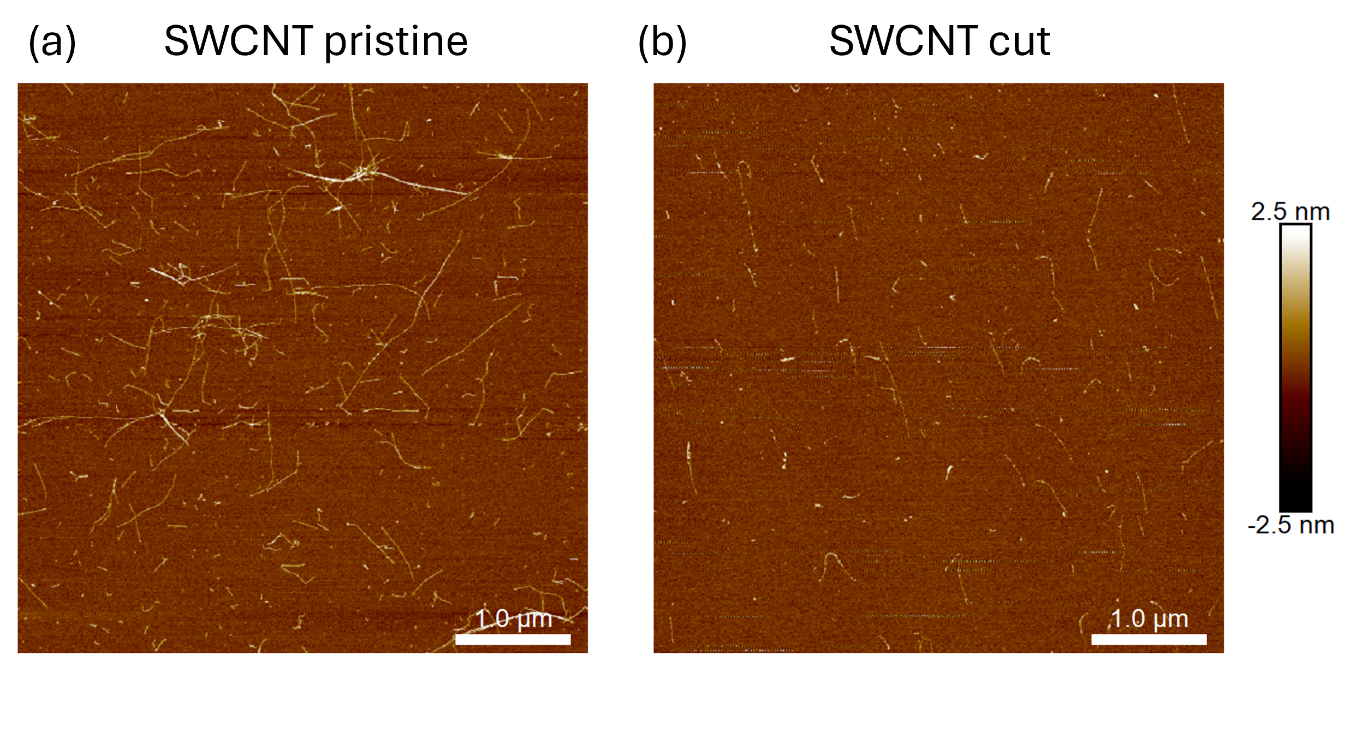


*Figure S5. Representative AFM images of (a) SWCNT pristine, (b) SWCNT cut, both after oxidation (Z-scale=5 nm).*


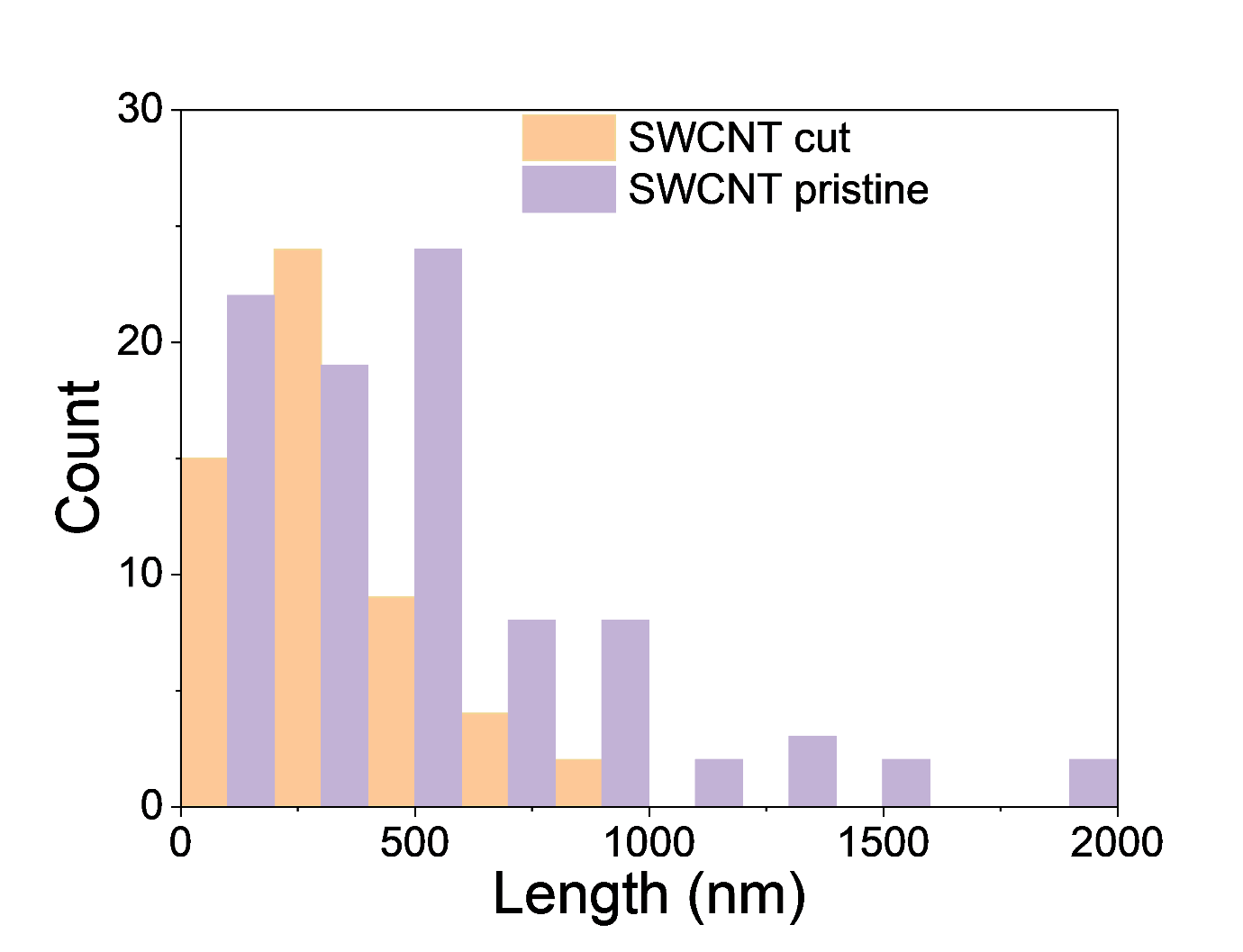


*Figure S6. Length distribution of pristine SWCNT (purple, median: 441nm, standard error of the mean (SEM): 26 nm) and oxidized SWCNT (yellow, median: 272 nm, SEM 41 nm), obtained from the analysis of 90 and 54 individual SWCNTs, respectively.*


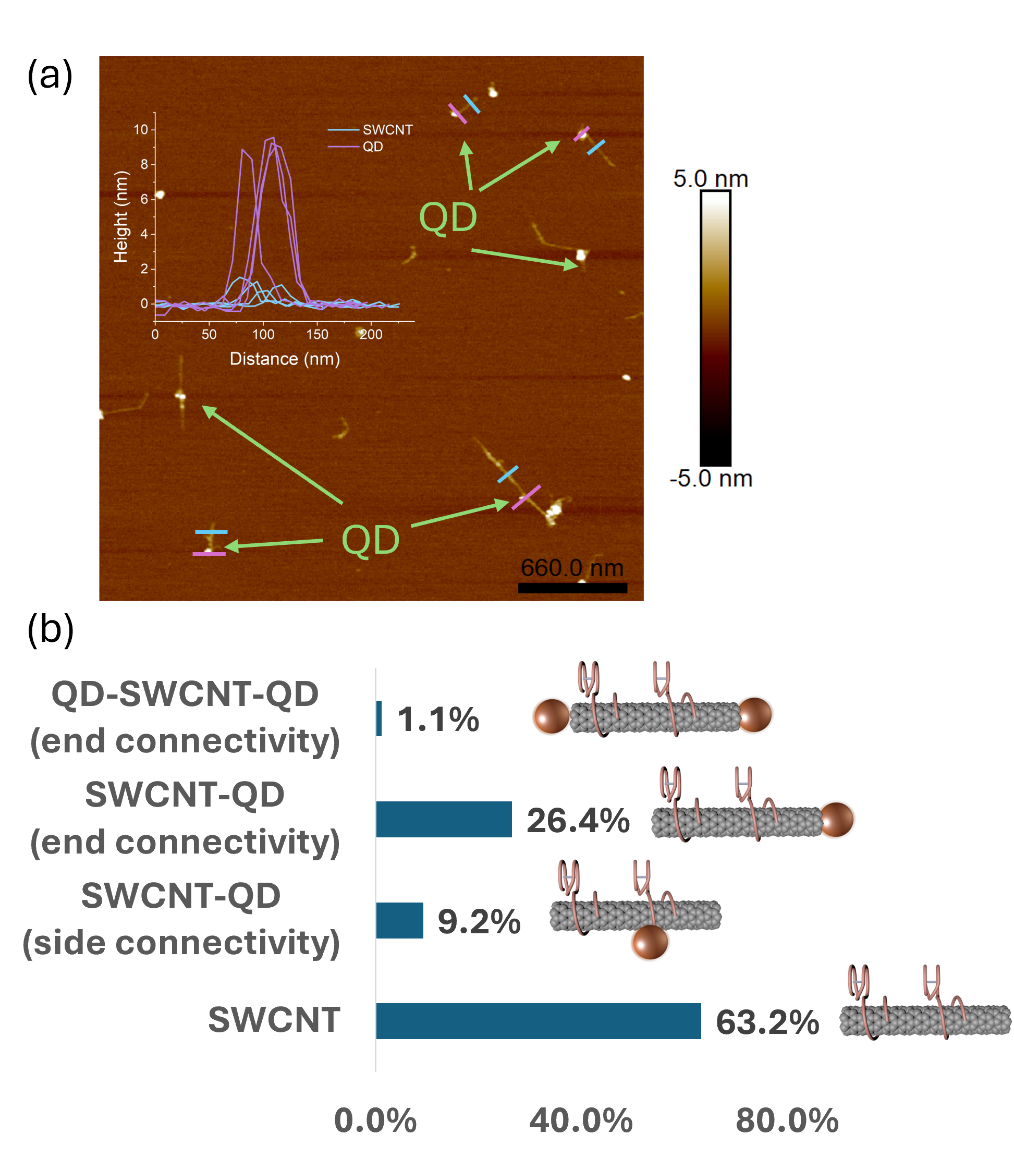


*Figure S7 (a) Representative AFM image of QD-SWCNT (Z-scale=10 nm), (b) Statistical distribution observed in the QD-SWCNT hybrids assembly, obtained from the analysis of 261 individual SWCNTs based on AFM imaging.*


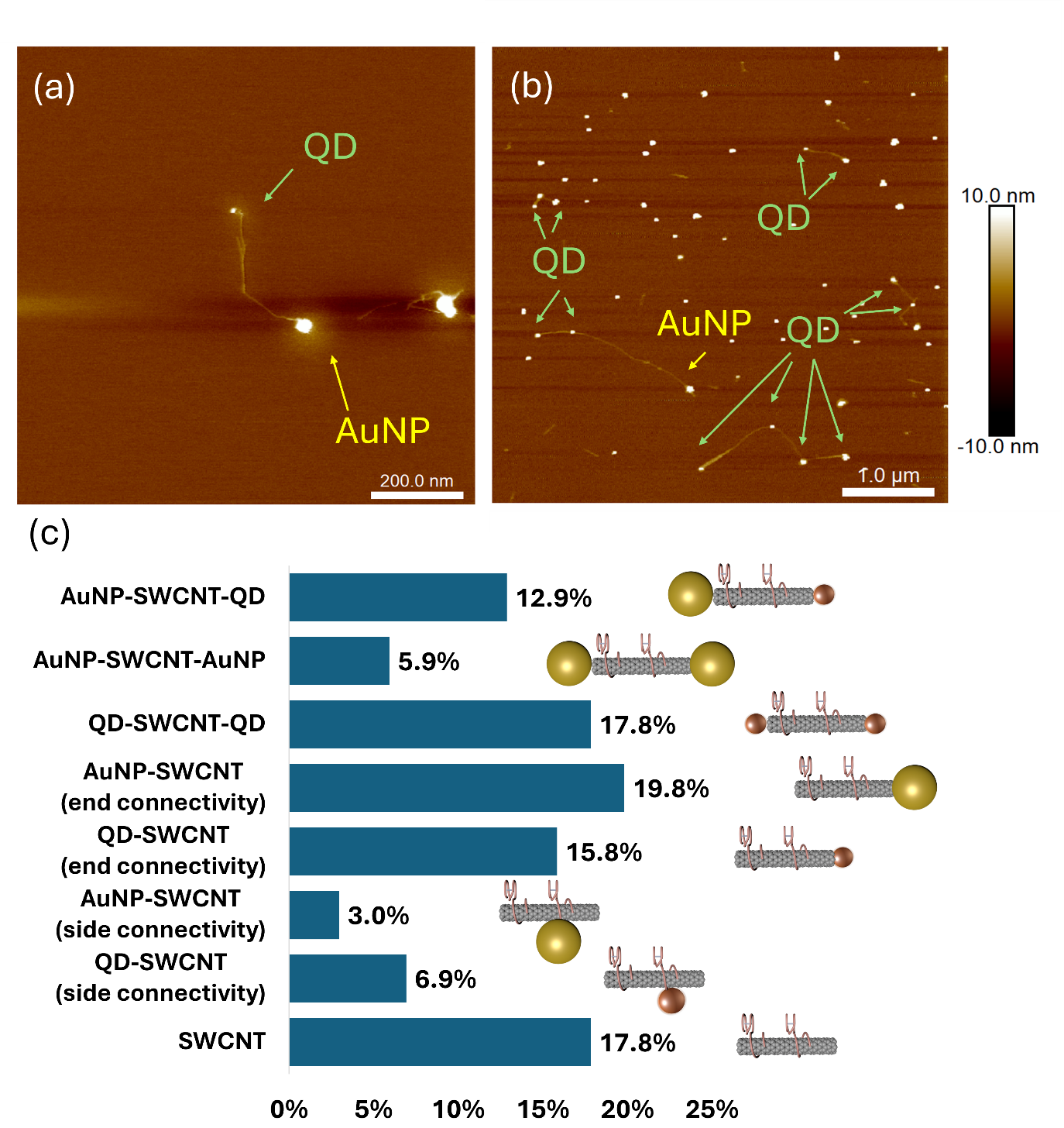


*Figure S8. Representative AFM images of (a) 1:1:1, (b) linear and branched AuNP-SWCNT-QD hybrids, QDs are highlighted with the green arrows and AuNPs are highlighted with the yellow arrows (Z-scale=20 nm). (c) Statistical distribution observed in the AuNP-SWCNT-QD hybrids assembly, obtained from the analysis of 101 individual SWCNTs based on AFM imaging (junction structures - e.g., QD-SWCNT-QD-SWCNT-AuNP- were counted as individual NP-SWCNT hybrids, i.e. as QD-SWCNT-QD and QD-SWCNT-AuNP). Notably, the higher proportion of QD–SWCNT–QD assemblies observed here compared to Figure S7 is likely due to the extended incubation time during the AuNP conjugation step, which allows excess free QDs to further react with available termini on the SWCNTs.*

*
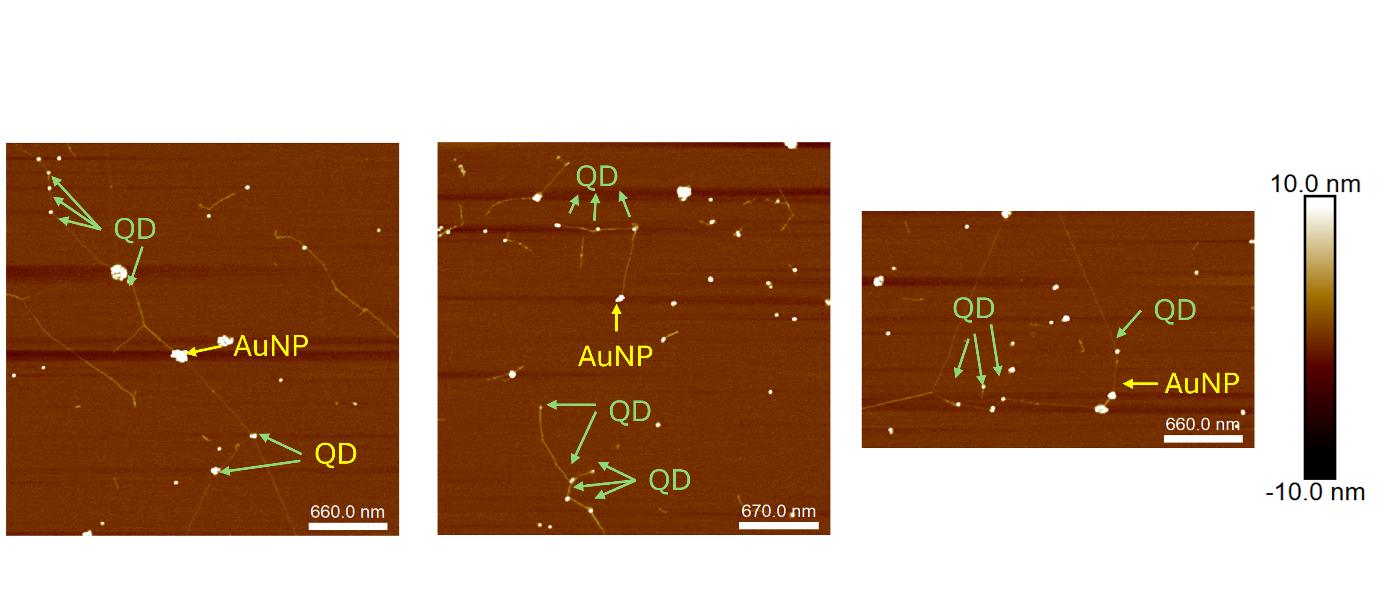
*

Figure S9. Representative AFM image of AuNP–SWCNT–QD heterostructures showing terminally functionalized nanotubes. Yellow arrows indicate AuNPs and green arrows indicate QDs conjugated at opposite termini of individual SWCNTs. The image highlights the successful formation of asymmetric nanohybrids in solution. Z-scale = 20 nm.


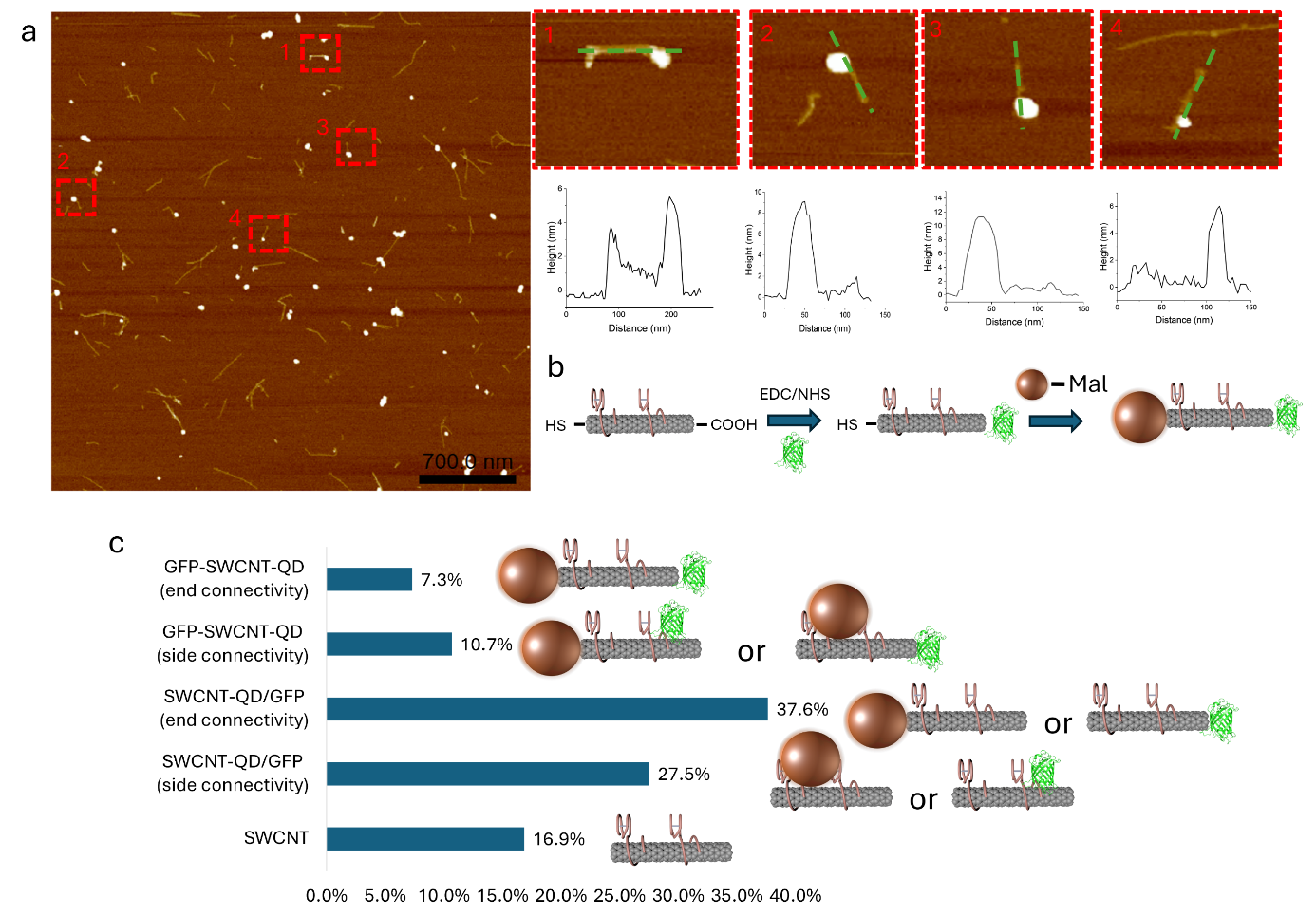


Figure S10.Characterization of GFP-SWCNT-QD heterostructures (from xxxx) . (a) Representative AFM image of GFP-SWCNT-QD hybrids. Selected hybrids are highlighted with red dashed boxes; a corresponding height profile is shown below (Z-scale = 20 nm). (b) Schematic illustration of the stepwise assembly process for the formation of terminally functionalized SWCNT nanohybrids. (c) Statistical distribution observed in the GFP-SWCNT-QD hybrid assembly, based on the analysis of 178 individual SWCNTs from multiple AFM images.

Nanoparticle Conjugation Conditions Optimization

**1. Buffer Selection and Composition Optimization**

**1.1 Tris–KCl Buffer System**

A buffer composed of Tris–HCl-KCl was selected as the primary medium for conjugation reactions and AFM sample preparation. This buffer offers several advantages:

- Electrostatic shielding, which enhances colloidal stability and reduces repulsion between negatively charged SWCNTs and nanoparticles
- Improved adsorption of the sample onto mica substrates
- Structural stabilization of the G-quadruplex domain in the DNA wrapping, critical for localized hemin binding

We also tested PBS as an alternative buffer system. However, during subsequent functionalization steps, PBS led to inconsistent hybrid formation and reduced binding to mica, and was thus excluded from further optimization.

**1.2 Tris-KCl/TBE Ratio Optimization**

We systematically varied the ratio of **Tris–KCl to TBE buffer** to evaluate its effect on hybrid formation and nanoparticle dispersion. The results are summarized below:

Table S1. Tris-KCl/TBE ratio optimization conditions.

| Sample | SWCNT (ng) | Tris-KCl (µL) | TBE (µL) | AuNP (µL, 1 OD) | Notes |
| --- | --- | --- | --- | --- | --- |
| HE-13 | 31.25 | 80 | 20 | 10 | Optimal |
| HE-14 | 31.25 | 40 | 20 | 10 |  |
| HE-15 | 31.25 | 60 | 30 | 10 |  |
| HE-16 | 31.25 | 40 | 10 | 10 |  |
| HE-17 | 31.25 | 20 | 20 | 10 |  |

As shown in Figure S17, the balance between Tris and TBE was found to be critical. Increasing the TBE content (as in HE-15) led to AuNP aggregation and formation of large, non-uniform SWCNT–Au clusters. At even higher TBE ratios, AuNP visibility under AFM was significantly reduced.

We propose two main explanations for this effect:

1. At low concentration, EDTA present in TBE may chelate cationic stabilizers (e.g., CTAB or metal ions) on AuNP surfaces, reducing their colloidal stability and promoting aggregation, further enhance the hybrid yield.
2. At hight concentration, EDTA, as a strong electrolyte, can raise the ionic strength of the solution, triggering nanoparticle precipitation and reducing hybrid formation efficiency.


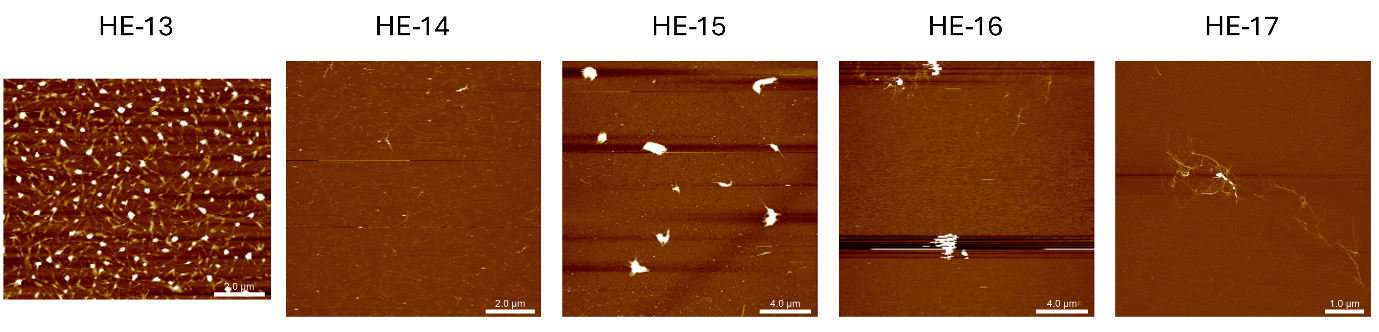


Figure S11. Representative AFM image of AuNP–SWCNT heterostructures formed under different Tris–KCl/TBE buffer ratios.

Thus, HE-13, with a Tris:TBE ratio of 4:1, was selected as the optimal buffer composition for hybrid conjugation and imaging.

**1.3 TCEP Addition for Thiol Activation**

To explore whether disulfide formation might limit Au–S coupling efficiency, we tested the use of TCEP (tris(2-carboxyethyl)phosphine) as a reducing agent prior to AuNP conjugation. However, no significant improvement was observed in hybrid yield or nanoparticle attachment efficiency, and this step was omitted in the final protocol.

2.**Alternative Nanoparticles: QD–Maleimide Conjugation**

To conjugate QDs to the thiol-terminated SWCNTs, we functionalized amine-modified QDs using sulfo-EMCS (Thermo, #22307), a heterobifunctional linker with NHS ester and maleimide groups.
Experimental procedure:

- Mix 2 µL of QD solution (1 mg/mL) with 2 µL of sulfo-EMCS (10 mM) in deionized water
- Incubate for 30 min at room temperature
- Purify the resulting maleimide-functionalized QDs (QD-mal) using Amicon Ultra centrifugal filters (30 kDa MWCO)

Hybrid formation between QD-mal and SWCNT–SH was tested across a range of QD concentrations:

Table S2. Optimization of QD-maleimide to SWCNT (thiol-end) ratio for terminal conjugation efficiency.

| Sample | SWCNT(ng) | Buffer (Tris-KCl/TBE 4:1, µL) | QD-mal (µL) | Notes |
| --- | --- | --- | --- | --- |
| HE-18 | 1.875 | 5 | 2 |  |
| HE-19 | 1.875 | 5 | 1 |  |
| HE-20 | 1.875 | 5 | 0.5 | Optimal |
| HE-21 | 1.875 | 5 | 0.2 |  |

AFM imaging (Figure S12) confirmed that **sample HE-20** yielded the highest proportion of distinct QD–SWCNT conjugates with minimal background aggregation. Figure S13 shows representative images and quantitative analysis of hybrid formation efficiency.


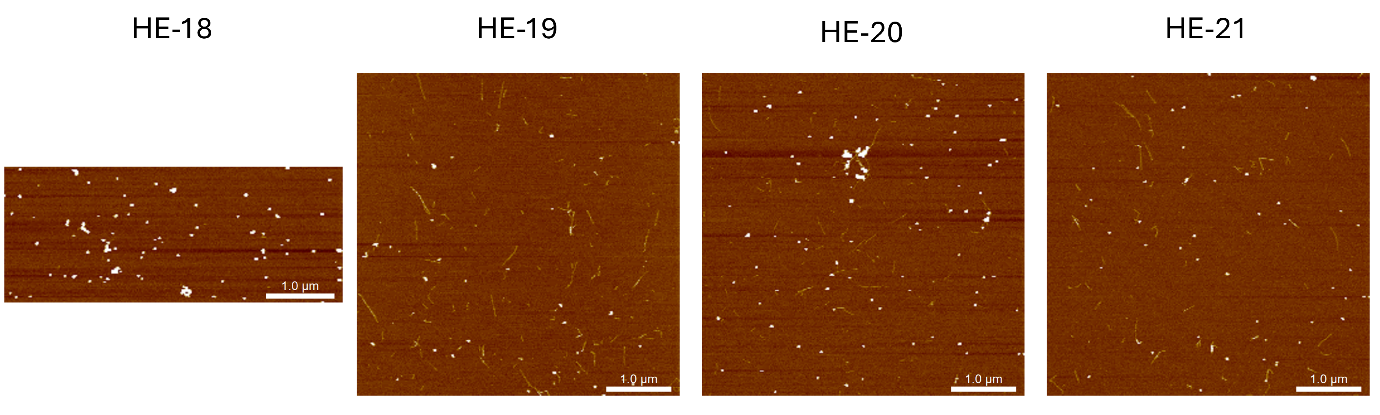


Figure S12. Representative AFM image of QD–SWCNT heterostructures formed under different QD-to-SWCNT ratios.


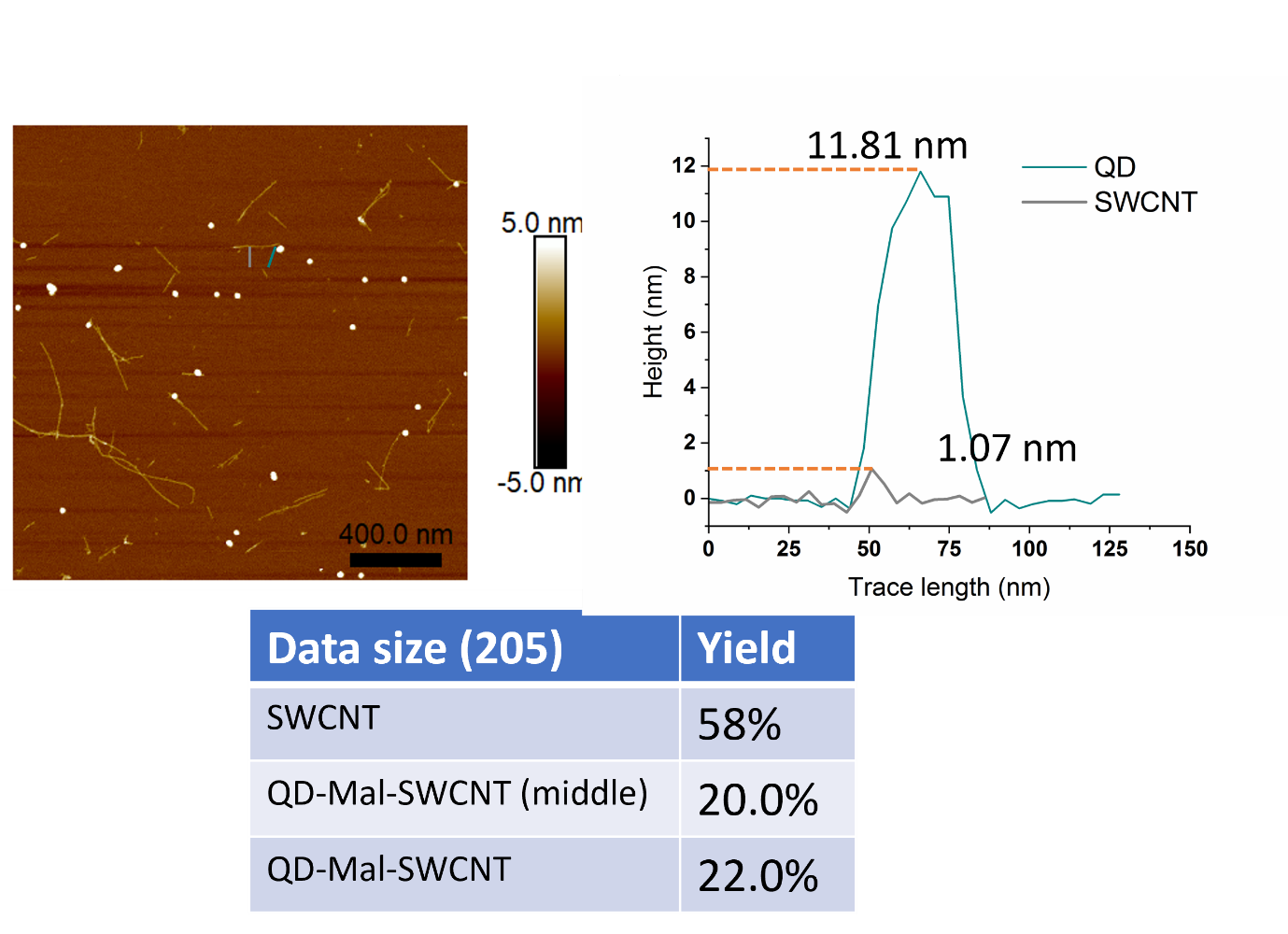


Figure S13. Representative AFM image of QD–SWCNT heterostructures (Z-scale = 10 nm), with a corresponding height profile along the cross-section indicated in the image. Statistical distribution of nanostructures observed in the QD–SWCNT, based on the analysis of 205 individual SWCNTs imaged by AFM.

**3. Optimization of Conjugation Ratios for QDs and GFP**

To determine the optimal ratios for coupling carboxyl-functionalized SWCNTs with amine-bearing particles (QDs and GFP), we performed a systematic screening using EDC/NHS chemistry.

**3.1 QD-to-SWCNT Ratio Optimization**

QD conjugation was carried out using varying dilutions of QDs (1 mg/mL stock), with fixed amounts of SWCNTs (1.875 ng) and EDC/NHS (50 mM/100 mM):

Table S3. Optimization of QD-to-SWCNT (carboxylic-end) ratio for terminal conjugation.

| Sample | SWCNT(ng) | EDC/NHS (50 mM/100mM) | QD dilution (µL, 1 mg/mL) | Notes |
| --- | --- | --- | --- | --- |
| HE-22 | 1.875 | 1 | 10^4 |  |
| HE-22 | 1.875 | 1 | 10^5 | Optimal |
| HE-22 | 1.875 | 1 | 10^6 | Optimal |
| HE-22 | 1.875 | 1 | 10^7 |  |

AFM imaging indicated that QD dilutions in the range of 10⁻⁵ to 10⁻⁶ provided optimal hybrid formation without excessive free QDs or aggregation

**3.2 GFP-to-SWCNT Ratio Optimization**

Similarly, different concentrations of GFP (5 µg/mL stock) were tested while keeping SWCNT and EDC/NHS amounts constant:

Table S4. Optimization of GFP-to-SWCNT (carboxylic-end) ratio for terminal conjugation.

| Sample | SWCNT(ng) | EDC/NHS (50 mM/100mM) | GFP (5µg/mL) | Notes |
| --- | --- | --- | --- | --- |
| HE-23 | 0.2 | 0.1 | 0.2 |  |
| HE-24 | 0.2 | 0.1 | 0.5 |  |
| HE-25 | 0.2 | 0.1 | 1 |  |
| HE-26 | 0.2 | 0.1 | 2 | Optimal |
| HE-27 | 0.2 | 0.1 | 4 | Optimal |

Table S5. Comparison of asymmetric SWCNT functionalization strategies across key parameters.

| **Method** | **SWCNT physical state** | **Site Specificity** | **Type of Functionalization** | **Scale** | **Moiety conjugation** | **Single-Molecule Control** | **Ref.** |
| --- | --- | --- | --- | --- | --- | --- | --- |
| Interfacial Inversion | Film | Moderate (film-level) | Covalent: UV cycloaddition, radical addition^[2]^; Non-covalent spin-coating^[3]^ | Film (µm scale) | Polymer^[3]^ | ⮽ | ^[2,3]^ |
| Electrochemical Polarization | Poorly dispersed (likely bundled) | High (on bundled termini) | Electrodeposition via electric field polarization | µm scale | Metal particles | ⮽ | ^[4,5]^ |
| Protection–Deprotection | Anchored to substrate | Low (lacking sidewall protection) | Covalent: esterification^[6]^; Non-covalent + amidation^[7]^ | Film (µm scale) | Metal^[6]^; metal and protein^[7]^ | ⮽ | ^[6,7]^ |
| **This work** | Fully dispersed in solution (via DNA) | High (individual CNT termini) | Covalent: UV cycloaddition and amidation | nm scale | Metal, semiconductor (QDs), and protein | ☑ |  |


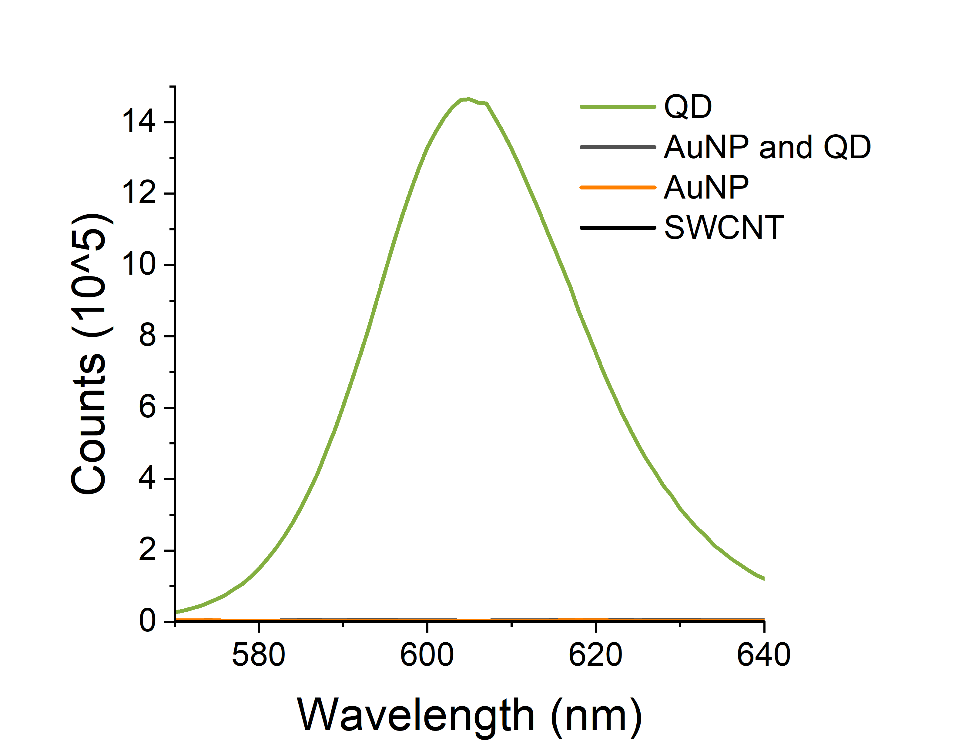


Figure S14. Steady-state photoluminescence characterization of QDs (green), QDs mixed with AuNPs (grey), AuNPs only (orange), and SWCNTs only (black) under 450 nm excitation.

*
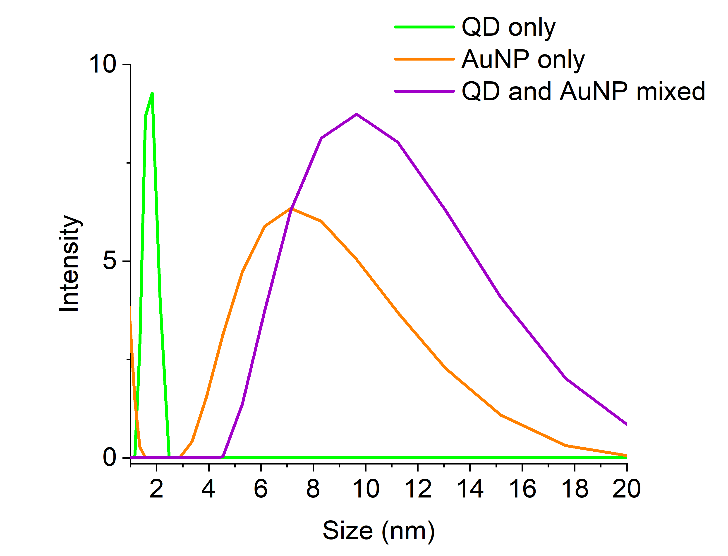
*

Figure S15. DLS measurements showing the hydrodynamic size distribution of QDs (green), AuNPs (orange), and the QD–AuNP mixture (purple).


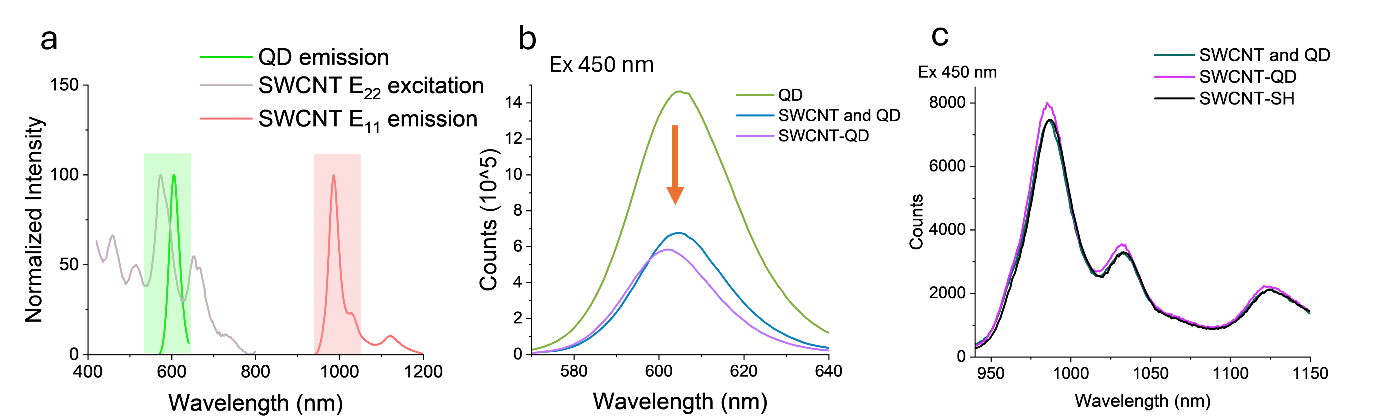


Figure S16. (a) Spectra of QDs emission (green), SWCNTs excitation (gray) and SWCNT emission (light red) showing spectral overlap that may permit energy transfer. Comparison of (b) QD and (c) SWCNT PL spectra under QD excitation conditions for QD alone, QD-SWCNT mixture (non-covalent), and covalent QD-SWCNT hybrids, indicating that PL enhancement occurs only in the covalent hybrids, furhter supporting charge transfer being the dominant mechanism.


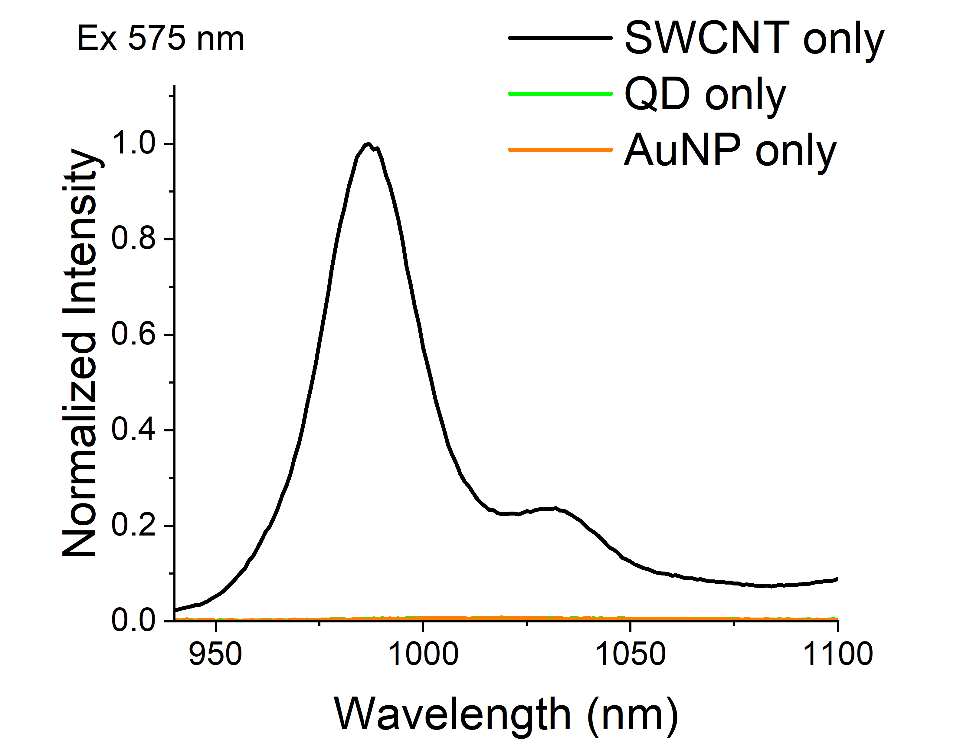


Figure S17. Steady-state photoluminescence characterization of QDs (green), AuNPs only (orange), and SWCNTs only (black) under 575 nm excitation.

*
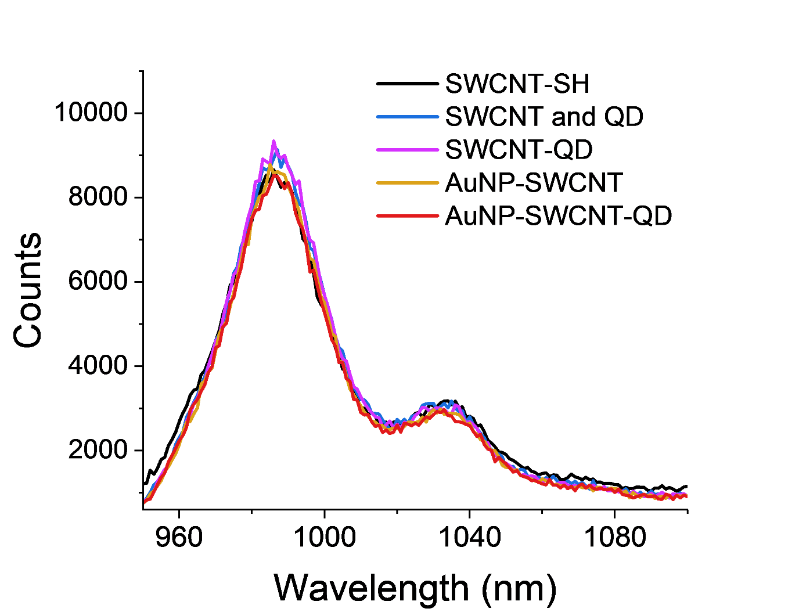
*

*Figure S18. Steady-state photoluminescence characterization under 845 nm excitation for thiol-functionalized SWCNT after cutting (black), control of SWCNT mixed with QD (blue), SWCNT-QD hybrids (purple), AuNP-SWCNT hybrids (yellow), AuNP-SWCNT-QD (red).*


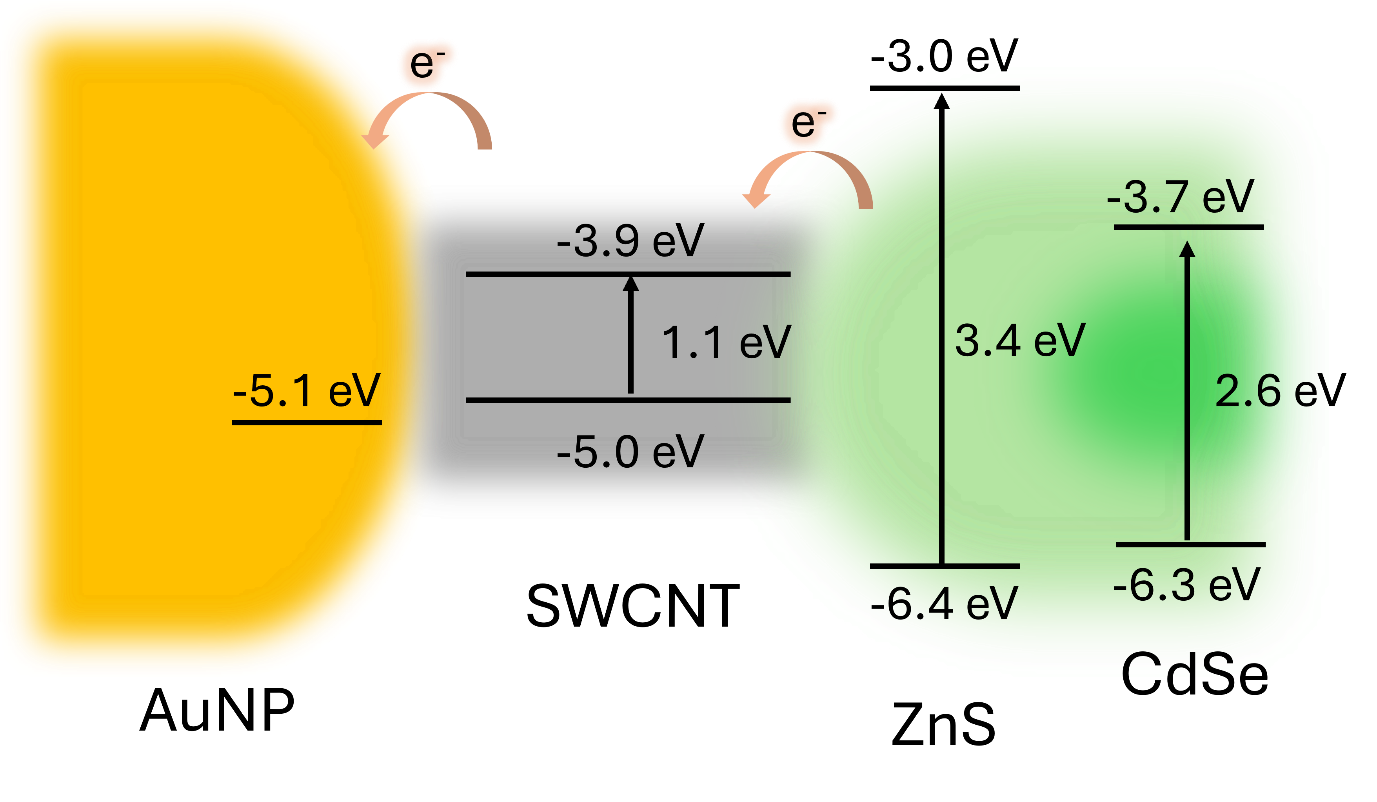


*Figure S19. Band gap energy and relative band edge locations for AuNP-SWCNT-QD heterostructures. The electron transfer reaction under illumination is indicated by arrows. The values of the work function are adapted from previous reports*^[8–10]^*.*


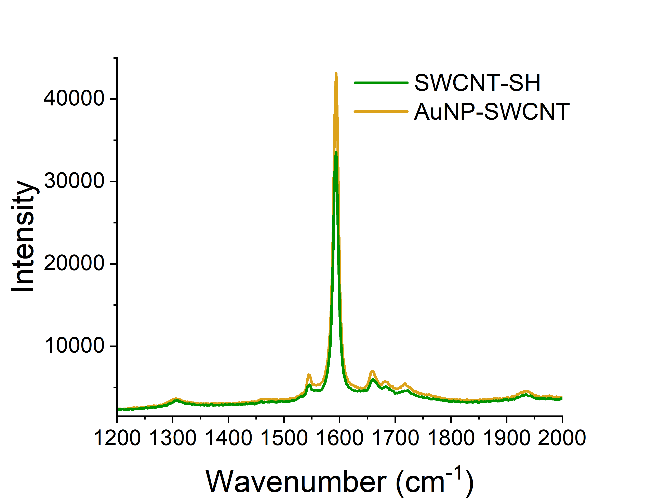


Figure S20. Raman spectra of thiol-functionalized SWCNTs (green) and AuNP-conjugated SWCNTs (yellow), measured under 633 nm excitation. The D/G ratios were found to be 0.08 and 0.07 respectively.


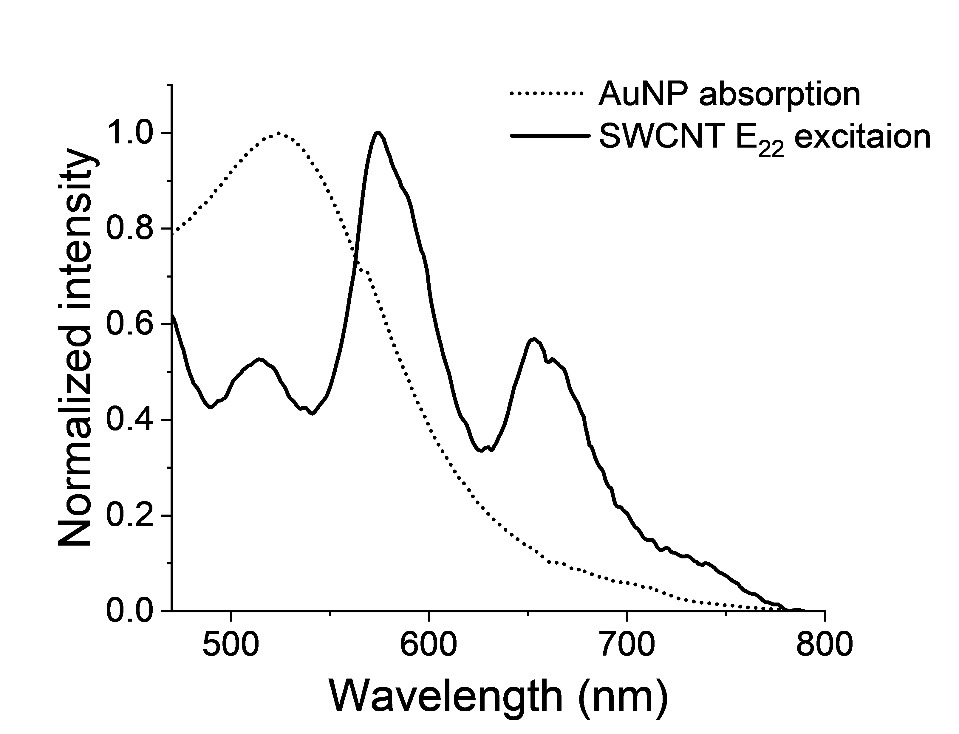


Figure S21.UV-Vis absorbance spectrum of AuNPs (dotted line) and SWCNT (black).

**References**

[1] H. Atsumi, A. M. Belcher, DNA Origami and G-Quadruplex Hybrid Complexes Induce Size Control of Single-Walled Carbon Nanotubes via Biological Activation, *ACS Nano* (2018), *12*, 7986, https://doi.org/10.1021/acsnano.8b02720.

[2] K. M. Lee, L. Li, L. Dai, Asymmetric End-Functionalization of Multi-Walled Carbon Nanotubes, *Journal of the American Chemical Society* (2005), *127*, 4122, https://doi.org/10.1021/ja0423670.

[3] S. Ozden, L. Ge, T. N. Narayanan, et al., Anisotropically Functionalized Carbon Nanotube Array Based Hygroscopic Scaffolds, *ACS Applied Materials & Interfaces* (2014), *6*, 10608, https://doi.org/10.1021/am5022717.

[4] C. Warakulwit, T. Nguyen, J. Majimel, et al., Dissymmetric Carbon Nanotubes by Bipolar Electrochemistry, *Nano Letters* (2008), *8*, 500, https://doi.org/10.1021/nl072652s.

[5] G. Loget, V. Lapeyre, P. Garrigue, et al., Versatile Procedure for Synthesis of Janus-Type Carbon Tubes, *Chemistry of Materials* (2011), *23*, 2595, https://doi.org/10.1021/cm2001573.

[6] Z. Wei, M. Kondratenko, L. H. Dao, D. F. Perepichka, Rectifying Diodes from Asymmetrically Functionalized Single-Wall Carbon Nanotubes, *Journal of the American Chemical Society* (2006), *128*, 3134, https://doi.org/10.1021/ja053950z.

[7] S. Mantha, B. Chin, A. L. Simonian, Electrochemical biosensing of organophosphates using vertically aligned multiwall carbon nanotubes, *2013 Seventh International Conference on Sensing Technology (ICST)* (2013), 71, https://doi.org/10.1109/ICSensT.2013.6727619.

[8] Z. Kuang, F. J. Berger, J. L. P. Lustres, et al., Charge Transfer from Photoexcited Semiconducting Single-Walled Carbon Nanotubes to Wide-Bandgap Wrapping Polymer, *The Journal of Physical Chemistry C* (2021), *125*, 8125, https://doi.org/10.1021/acs.jpcc.0c10171.

[9] R. C. Pawar, S. Kang, S. H. Ahn, C. S. Lee, Gold nanoparticle modified graphitic carbon nitride/multi-walled carbon nanotube (g-C3N4/CNTs/Au) hybrid photocatalysts for effective water splitting and degradation, *RSC Advances* (2015), *5*, 24281, https://doi.org/10.1039/C4RA15560B.

[10] Y. Li, N. Chopra, Fabrication of nanoscale heterostructures comprised of graphene-encapsulated gold nanoparticles and semiconducting quantum dots for photocatalysis, *Physical Chemistry Chemical Physics* (2015), *17*, 12881, https://doi.org/10.1039/C5CP00928F.
